# An annotated catalogue of type specimens of the land snail genus *Cyclophorus* Monfort, 1810 (Caenogastropoda, Cyclophoridae) in the Natural History Museum, London

**DOI:** 10.3897/zookeys.411.7258

**Published:** 2014-05-23

**Authors:** Nattawadee Nantarat, Chirasak Sutcharit, Piyoros Tongkerd, Jonathan Ablett, Fred Naggs, Somsak Panha

**Affiliations:** 1 Animal Systematics Research Unit, Department of Biology, Faculty of Science, Chulalongkorn University, Bangkok 10330, Thailand; 2 Biological Sciences Program, Faculty of Science, Chulalongkorn University, Bangkok 10330, Thailand; 3 Division of Higher Invertebrates, Natural History Museums, London, SW7 5BD, United Kingdom

**Keywords:** Gastropoda, type specimens, biohistory, NHM, taxonomy, land snails, Cyclophoridae, *Cyclophorus*

## Abstract

The collection of land caenogastropod snails in the genus *Cyclophorus* Monfort, 1810 housed in the Natural History Museum, London (NHM), includes 52 type lots. Lectotypes have been designated for 43 available species-level names to stabilize existing nomenclature, two previously designated lectotype, two holotypes, one paratype, one syntype, one possible syntype and two paralectotypes are also listed. A complete catalogue of the *Cyclophorus* types in NHM, London is provided for the first time.

## Introduction

The Cyclophoridae Gray, 1847 are a family of caenogastropod land snails with a fossil history dating back to the Early Tertiary (Gordon and Olson 1995). Extant cyclophorids are distributed in Africa, Asia, Australia, Southern Europe and various Pacific islands (Kobelt 1902, 1907–1908, Solem 1959, Stanisic 1998). Kobelt (1902, 1907–1908) carried out detailed reviews of the extensive nineteenth century literature on the Cyclophoroidea (= Cyclophoridae sensu Kobelt 1902); his work remains the standard reference for the group based on shell morphology. Subsequent work combined shell morphology with soft body anatomy, but intra- and interspecific variation in shell morphology within the Cyclophoridae, combined with a highly conserved soft body anatomy, resulted in little progress (Tielecke 1940, Andrews and Little 1972, Stanisic 1998, Barker 2001). Recent classifications of the Cyclophoridae have recognised three subfamilies: Cyclophorinae, Alycaeinae, and Spirostomatinae (Bouchet and Rocroi 2005). Thirty five genera containing approximately 810 species have been recognized in the Cyclophoridae (Kobelt 1902, 1907–1908, Wenz 1938, Vaught 1989, Bouchet and Rocroi 2005, Lee et al. 2008).

With about 22% of the species, *Cyclophorus* Monfort, 1810 is the most species rich genus in the Cyclophoridae amounting to about 180 nominal species (Kobelt 1902, 1908). *Cyclophorus* is distributed through the humid or seasonally humid tropical and warm temperate habitats of South Asia and SE Asia, including the southern areas of China, Korea and Japan (Kobelt 1902, 1907–1908, Gude 1921, Pilsbry 1926, Benthem Jutting 1948, 1949, Solem 1959, Minato and Habe 1982). Kobelt (1902) divided *Cyclophorus* into eight subgenera using shell size, shell shape, features of the peristome, umbilicus, and their geographical distribution. Subsequently Wenz (1938) recognized seven subgenera and then Vaught (1989) recognized only six subgenera. *Cyclophorus* species limits are generally poorly established. Some attempts have been made to provide a more secure basis for recognizing species limits using soft body anatomy and cytogenetic analysis (Welber 1925, Tielecke 1940, Andrews and Little 1972, Kasinathan 1975, Kongim et al. 2006). Most recently, Nantarat et al. (2014) clarified some relationships and species limits within *Cyclophorus* by using DNA sequences and constructing molecular trees.

*Cyclophorus* species level taxa were described solely on the basis of shell morphology and most of them were described without illustrations or designation of holotypes. The most prolific author of *Cyclophorus* species was O.F. Möllendorff, who described about 14% (26 taxa) of all *Cyclophorus* taxa. His type specimens housed in the Forschungsinstitut und Naturmuseum Senckenberg, Frankfurt were catalogued and illustrated by Zilch (1956). The other major contributors to descriptions of *Cyclophorus* species were L. Pfeiffer, H.H. Godwin-Austen, G.B. Sowerby I. and E.A. Smith. Most of the *Cyclophorus* species that they described are housed in the Natural History Museum, London (hereafter the Museum or NHM) and account for about 30% (58 taxa) of the *Cyclophorus* taxa (Kobelt 1902, 1907–1908, Gude 1921, Pilsbry 1926, Benthem Jutting 1948, 1949, Solem 1959, 1966, Minato and Habe 1982).

The Museum holds one of the largest collections of Mollusca in the world, rich in type specimens it is also one of the most important natural history collection. Dating back to 1753 the collections abound with historical material (MacLellan and Way 2012). As with any museum collection with a long history, the documentation of specimens is sometimes in a poor state and records may contain conflicting information such that the recognition of some type material is problematic. We have critically evaluated the type status of material by comparing specimens, labels with the specimens, information in the registers, and information provided in the original descriptions. Of notable value are the distinctive handwritten labels of Pfeiffer and ‘MC’, indicating that the lot was part of the Hugh Cuming collection (Breure and Ablett 2011). Syntype status can be established with different degrees of confidence, largely depending on the quality of information on specimen labels and information provided in the registers. Labels handwritten by the original author verifying type status appear to provide unequivocal evidence but there remained a possibility that specimens could have been mixed up and placed with the wrong labels and all specimens selected as lectotypes were carefully compared with the original description, original figures when available, and with any measurements provided in the original description. Type localities are cited as in original descriptions. Additional information from labels, current political boundaries or subsequently published localities is given in square brackets.

## Method

Specimens were photographed showing apertural, apical and umbilical views. Shell measurements were made for the adult specimen using digital calipers. The adult shell specimens are easy to be distinguished from juveniles by performing expanded and reflexed apertural lips. The specimen was measured accurately to 0.1 mm, and the expanded lip of aperture was included. Shell height (H) was measured along the columellar axis passing through apex to apertural base. Shell width (W) is the maximum width perpendicular to columella axis (Cox 1960: fig. 80). Numbers of whorl were counted, from the shell apex where incidence of the spiral sculpture approaches 90° as follow Burch and Pearce (1990: fig. 6).

**Abbreviations:** Material was examined from the following institutions: NHM, The Natural History Museum, London (NHM registered specimens are cited as NHMUK); MCZ, Museum of Comparative Zoology, Harvard University, Cambridge. Others abbreviations used are: D, shell diameter; H, shell height; W, number of whorls.

## Catalogue of the type specimens of *Cyclophorus* Montfort, 1810

### Cyclophorus aborensis

Godwin-Austen, 1915

http://species-id.net/wiki/Cyclophorus_aborensis

Cyclophorus aborensis Godwin-Austen, 1915: 494, pl. 38, fig. 1. Gude 1921: 69

#### Type locality.

Rotung, 2000 ft., near Egar stream; Kalek and Renging, 2000 ft.

#### Type material.

Lectotype (design. n.), NHMUK 1903.7.1.3051 from Renging (Fig. 2A; D=52.5 mm, H=40.2 mm, W=5), paralectotypes NHMUK 1903.7.1.3048 from Kalek (2 shells; Fig. 2B; D=49.1 mm, H=34.0 mm, W=5; D=48.6 mm, H=36.0 mm, W=5). NHMUK 1903.7.1.3049 from Rami Dambang, Abor (2 shells; D=33.0 mm, H=24.4 mm, W=5; D=29.6 mm, H=21.8 mm, W=5).

#### Remarks.

Godwin-Austen clearly states that this taxon was based on five lots of specimens from various localities. The original description included illustrations of two specimens from different localities, but only one set of measurement was given. In addition, the author clearly stated that two lots of the type series were kept in the Indian Museum, and the remaining three type lots were housed in the NHM. The specimen NHMUK 1903.7.1.3051 with Godwin-Austen handwriting label stating “Co-type” and figured in the original description (Godwin-Austen 1915: figs 1, 1a) is here designated as the lectotype. The other 2 specimen lots housed in the NHM (nos. 3048 and 3049), and the two lots previously housed in the Indian Museum, that were transferred to the Zoological Survey of India (nos. 6009 and 6010), are therefore paralectotypes. Following our lectotype designation, the type locality of this species is fixed as Renging, Abor Hills (altitude 2000 ft.) (ICZN 1999: Art. 76.2).

### Cyclophorus affinis

Theobald, 1858

http://species-id.net/wiki/Cyclophorus_affinis

Cyclophorus affinis Theobald, 1858: 246. Hanley and Theobald 1870: 1, 28, pl. 2, fig. 7 and pl. 48, fig. 2. Kobelt 1902: 135. Kobelt 1908: 654.

#### Type locality.

Maulmein [Mawlamyine, Myanmar].

#### Type material.

Lectotype (design. n.), NHMUK 1903.7.1.1454 (Fig. 2C; D=34.9 mm, H=28.7 mm, W=5).

#### Remarks.

The original description stated this taxon was based on two individuals, and gave the dimensions of both specimens. The single specimen from the Godwin-Austen collection, purchased from Theobald, closely matches the larger of the two measurements given in the original description and figured in Hanley and Theobald (1870: pl. 2, fig. 7). This specimen is designated as the lectotype. The type specimen relating to the smaller of the two measurements could not be located in the NHM collections.

### Cyclophorus amoenus

(Pfeiffer, 1854)

http://species-id.net/wiki/Cyclophorus_amoenus

Cyclostoma (Cyclophorus) amoenum Pfeiffer, 1854b [1852]: 62. Pfeiffer 1854a: 346, pl. 45, figs 11, 12.
Cyclophorus amoenus – Reeve, 1861: sp. 40. Kobelt 1902: 97.

#### Type locality.

unknown.

#### Type material.

Lectotype (design. n.), NHMUK 20130113/1 (Fig. 3A; D=30.0 mm, H=23.5 mm, W=4½), paralectotype NHMUK 20130113/2 (1 shell; Fig. 3B; D=26.0 mm, H=22.5 mm, W=4½).

#### Remarks.

This species was described based on specimens from the Cuming collection. In the original description only one set of specimen measurements was given. In 1854, Pfeiffer (1854a: 346, pl. 45, figs 11, 12) re-published the description and illustrated a single specimen from the Cuming collection. There are two specimens from the Cuming collection with an original label in Pfeiffer’s handwriting stating the species name, and a subsequent label stating “Type”. The figured specimen in Pfeiffer (1854a) can be recognized by the varix on the last whorl, and is designated here as the lectotype.

### Cyclophorus appendiculatus

(Pfeiffer, 1854)

http://species-id.net/wiki/Cyclophorus_appendiculatus

Cyclostoma (Cyclophorus) appendiculatum Pfeiffer, 1854b [1852]: 61. Pfeiffer 1854a: 345, pl. 45, figs 7, 8.
Cyclophorus appendiculatus – Kobelt, 1902: 106. Kobelt 1907: 584.

#### Type locality.

Insulis Philippinis [Philippines].

#### Type material.

Lectotype (design. n.), NHMUK 20130079/1 (Fig. 3C; D=34.3 mm, H=20.1 mm, W=4½), paralectotypes NHMUK 20130079/2-3 (2 shells, Fig. 3D; D=32.1 mm, H=19.8 mm, W=4½ and D=34.4 mm, H=21.8 mm, W=4½).

#### Remarks.

This species was described based on specimens from the Cuming collection. In the original description only one set of specimen measurements was given. In 1854, Pfeiffer (1854a: 345, pl. 45, figs 7, 8) re-described and illustrated a single specimen from the Cuming collection. There are three specimens from the Cuming collection with an original label in Pfeiffer’s handwriting stating the species name. The figured specimen in Pfeiffer (1854a) closely matches the measurements given in the original description and is here designated as the lectotype.

### Cyclophorus aquilus

(Sowerby I, 1843)

http://species-id.net/wiki/Cyclophorus_aquilus

Cyclostoma aquilum Sowerby I, 1843b: 61 [March]. Sowerby I 1843c: 123, pl. 27, fig. 131 [June]. Pfeiffer 1846: 14, pl. 8, figs 1, 2.
Cyclophorus aquilus – Reeve 1861: sp. 45. Kobelt 1902: 124. Kobelt 1907: 578.

#### Type locality.

Singapore.

#### Type material.

Lectotype (design. n.), NHMUK 20110225/1 (Fig. 3E; D=39.9 mm, H=31.7 mm, W=5), paralectotypes NHMUK 20110225/2-3 (2 shells; Fig. 3F; D=38.7 mm, H=29.5 mm, W=5, and D=39.9 mm, H=31.2 mm, W=5).

#### Remarks.

A figure was not provided in the original description but a figure in “Thes. Conch. part 3, pl. 27, fig. 131” was cited. The original description states ‘Found in the Woods at Singapore under decayed leaves by H. Cuming’. There are three specimens from the Cuming collection with an original label, possibly in Sowerby’s handwriting that states ‘Singapore in the wood under decayed leaves’. We therefore recognize these specimens as syntypes and the figured specimen (Sowerby I 1843c: pl. 27, fig. 131) is here designated as the lectotype.

### Cyclophorus bapuensis

Godwin-Austen, 1915

http://species-id.net/wiki/Cyclophorus_bapuensis

Cyclophorus (Glssostylus) bapuensis Godwin-Austen, 1915: 494, 495, pl. 38, fig. 2. Gude 1921: 57.

#### Type locality.

Abor Hills, vicinity of Bapu. [Arunachal Pradesh, India].

#### Type material.

Lectotype (design. n.), NHMUK 1903.7.1.3108/1 (Fig. 4A; D=32.3 mm, H=23.7 mm, W=5), paralectotypes NHMUK 1903.7.1.3108/2-3 (2 shells; Fig. 4B; D=30.3 mm, H=22.3 mm, W=5, and D=30.0 mm, H=22.4 mm, W=5).

#### Remarks.

The original description stated “Type no. 3108 Brit. Mus.” There are three specimens in the type lot number 3018 from the Godwin-Austen collection. The specimen figured in the original description corresponds to the measurements given in the original description and is here designated as the lectotype.

### Cyclophorus beddomeanus

Preston, 1914

http://species-id.net/wiki/Cyclophorus_beddomeanus

Cyclophorus beddomeanus Preston, 1914: 21, text-fig. Gude 1921: 74, 75.

#### Type locality.

Naga Hills [Assam, Arunachal Pradesh and Nagaland, India].

#### Type material.

Lectotype (design. n.), NHMUK 1936.4.15.22 (Fig. 4C; D=53.8 mm, H=44.0 mm, W=5).

#### Remarks.

The original description gives a range of shell dimensions so this species is clearly based on more than one specimen. Preston mentions a “white-lipped variety” that was included in the type series but which could not be located in the NHM collections. The specimen 1936.4.15.22 is the one figured in the original description and is designated as the lectotype.

### Cyclophorus bensoni

(Pfeiffer, 1854)

http://species-id.net/wiki/Cyclophorus_bensoni

Cyclostoma bensoni Pfeiffer, 1854c [1852]: 158. Pfeiffer 1853a: 244, pl. 32, figs 11–13.
Cyclophorus bensoni – Hanley and Theobald 1870: 16, pl. 34, fig. 5. Kobelt 1902: 108.

#### Type locality.

unknown.

#### Type material.

Lectotype (design. n.), NHMUK 20130115 (Fig. 4D; D=42.8 mm, H=35.5 mm, W=5).

#### Remarks.

This species was described based on specimens from the Cuming collection. In the original description only one set of specimen measurements was given. In 1853, Pfeiffer (1853a: 244, pl. 32, figs 11–13) re-described and illustrated a single specimen from the Cuming collection. There is a single specimen from the Cuming collection with an original label in Pfeiffer’s handwriting stating the species name. The figured specimen in Pfeiffer (1853a) closely matches the measurements given in the original description and is here designated as the lectotype.

### Cyclophorus cochranei

Godwin-Austen, 1889

http://species-id.net/wiki/Cyclophorus_cochranei

Cyclophorus cochranei Godwin-Austen, 1889: 334, 335. Smith 1895: 119, pl. 4, fig. 2. Kobelt 1902: 127.

#### Type locality.

Niah Hills [Sarawak, Malaysia].

#### Type material.

Lectotype (design. n.), NHMUK 1889.12.7.5 (Fig. 5A; D=40.8 mm, H=29.9 mm, W=5).

#### Remarks.

In the original description, Godwin-Austen states there were three specimens, one from Niah Hills and two from Busan Hills. The NHM collections contain a single specimen from Busan Hills with a “T.” written on the shell. It matches the measurements given in the original description and is designated as the lectotype. The two other specimens (one from Busan, one from Niah Hills) could not be located in the NHM collections.

### Cyclophorus consociatus

Smith, 1893

http://species-id.net/wiki/Cyclophorus_consociatus

Cyclophorus consociatus Smith, 1893: 13. Kobelt 1902: 109.

#### Type locality.

Annam [Central Vietnam].

#### Type material.

Lectotype (design. n.), NHMUK 1893.2.26.8 (Fig. 5B; D=39.0 mm, H=34.0 mm, W=5), paralectotypes NHMUK 1893.2.26.9-10 (2 shells; Fig. 5C; D=36.9 mm, H=31.0 mm, W=5, and D=26.1 mm, H=21.8 mm, W=5).

#### Remarks.

There are three specimens in the type lot with original labels in Smith’s handwriting including a label stating “type”, and subsequently changed to read “holotype red spot”. The original description gives the measurements of only one shell which matches those of the NHM specimen with the red spot. This specimen is designated as the lectotype.

### Cyclophorus crassalabella

Godwin-Austen, 1888

http://species-id.net/wiki/Cyclophorus_crassalabella

Cyclophorus crassalabella Godwin-Austen, 1888: 244. Kobelt 1902: 110.

#### Type locality.

Shan Hills [Shan State, Myanmar].

#### Type material.

Lectotype (design. n.), NHMUK 1911.6.10.8 (Fig. 5D; D=41.5 mm, H=30.7 mm, W=5).

#### Remarks.

The original description gives the measurements of only one shell which matches those of the NHM specimen. The original label also states “P.Z.S. 1888 TYPE” in Godwin-Austen’s hand writing. This specimen is designated as the lectotype.

### Cyclophorus cucullata

(Gould, 1856)

http://species-id.net/wiki/Cyclophorus_cucullata

Cyclostoma cucullata Gould, 1856: 14.
Cyclophorus cucullatus – Reeve 1861: sp.44. Kobelt 1902: 127. Johnson 1969: 63.

#### Type locality.

Mergui Archipelago [Myanmar].

#### Type material.

Lectotype MCZ 169108, paralectotypes MCZ 169109 (1 shell) and NHMUK 20130116 (2 shells; Fig. 6A, B; D=27.2 mm, H=20.1 mm, W=5; D=26.5 mm, H=21.8 mm, W=5).

#### Remarks.

Johnson (1964: 63) stated “holotype, MCZ 169108”, which we consider to be a valid inadvertent lectotype designation (ICZN 1999: Art. 74.5). Pfeiffer (1858: 44) provided the description of this species based on Cuming collection material which he listed as “*Cyclostoma cucullata* Gould, MS” indicating that specimens under the manuscript name were presented to Cuming by Gould. In addition, Johnson (1964) confirms Gould met Cuming around 1857 leaving some specimens of his new species with Cuming. Later, Reeve (1861: sp. 44) re-published Pfeiffer’s description, and included illustrations of a specimen from the Cuming collection. There are two specimens in the NHM, one is the shell figured in Reeve (1861: sp. 44), with an original label stating “type”, and the locality is given as “Mergui Archipelago” Therefore, the NHM specimens received from Gould forms part of the type series and are paralectotypes.

### Cyclophorus eudeli

Smith, 1893

http://species-id.net/wiki/Cyclophorus_eudeli

Cyclophorus eudeli Smith, 1893: 13. Kobelt 1902: 137.

#### Type locality.

Annam [Central Vietnam].

#### Type material.

Lectotype (design. n.), NHMUK 1893.2.26.5 (Fig. 6B; D=40.0 mm, H=33.0 mm, W=5½), paralectotypes NHMUK 1893.2.26.6-7 (2 shells: 1 adult and 1 juvenile; Fig. 6C; D=40.1 mm, H=32.1 mm, W=5½).

#### Remarks.

The original description was clearly based on more than one specimen since it states “in examplis” (“in examples”) although only one set of shell measurements was given. There are three specimens in the NHM lot with an original label in Smith’s handwriting. The specimen that most closely matches measurements given in the description is designated as the lectotype.

### Cyclophorus everetti

Smith, 1892

http://species-id.net/wiki/Cyclophorus_everetti

Cyclophorus everetti Smith, 1892: 343, pl. 25, fig. 5. Kobelt 1902: 128. Kobelt 1908: 688.

#### Type locality.

Barit Mountain [north of Borneo, Malaysia].

#### Type material.

Lectotype (design. n.), NHMUK 1892.7.20.103 (Fig. 6D; D=36.7 mm, H=20.1 mm, W=4½), paralectotypes NHMUK 1892.7.23.1-2 (2 shells: 1 adult and 1 juvenile; Fig. 6E; D=33.6 mm, H=20.2 mm, W=4½).

#### Remarks.

Vermeulen (1999: 141) noted that the type specimens would be housed in the NHM, London but that he had not seen the specimens. We found four specimens of this species from Everett’s collection with original labels in Smith’s handwriting. This lot contained a juvenile specimen as was indicated in the original description. The figured specimen matches with the single set of shell measurements given in the original description and is designated as the lectotype.

### Cyclophorus exaltatus

(Pfeiffer, 1855)

http://species-id.net/wiki/Cyclophorus_exaltatus

Cyclostoma (Cyclophorus) exaltatum Pfeiffer, 1855b [1854]: 300.
Cyclophorus exaltatus – Kobelt 1902: 138. Kobelt 1908: 625.

#### Type locality.

Hong Kong, China.

#### Type material.

Lectotype (design. n.), NHMUK 1980041/1 (Fig. 7A; D=25.1 mm, H=25.3 mm, W=5), paralectotypes NHMUK 1980041/2-3 (2 shells; Fig. 7B; D=24.6 mm, H=24.4 mm, W=5 and D=23.3 mm, H=23.4 mm, W=5).

#### Remarks.

The NHM type lot was collected by Mr. Fortune and is from Cuming’s collection as stated in the original description. It has an original label in Pfeiffer’s handwriting giving the species name and collection locality. The specimen that most closely matches measurements given in the original description is here designated as the lectotype.

### Cyclophorus excellens

(Pfeiffer, 1855)

http://species-id.net/wiki/Cyclophorus_excellens

Cyclostoma (Cyclophorus) excellens Pfeiffer, 1855a [1854]: 126, 127.
Cyclophorus excellens – Pfeiffer 1854e: 11, pl. 4, figs 1, 2. Kobelt 1902: 128. Kobelt 1908: 670.

#### Type locality.

Unknown.

#### Type material.

Lectotype (design. n.), NHMUK 20130084/1 (Fig. 7C; D=52.5 mm, H=35.9 mm, W=5), paralectotype NHMUK 20130084/2 (1 shell; Fig. 7D; D=44.0 mm, H=31.3 mm, W=5).

#### Remarks.

This species was described based on material from the Cuming collection, and only one set of shell measurement was given. Later, Pfeiffer (1854e: pl. 4, figs 1, 2) re-published the description and figured a shell from the Cuming collection. Two shells from Cuming’s collection with an original label in Pfeiffer’s handwriting giving the species name are in the NHM collections. The specimen which most closely matches the measurements given in the original description and the illustration in Pfeiffer (1854e) is here designated as the lectotype.

### Cyclophorus expansus

(Pfeiffer, 1853)

http://species-id.net/wiki/Cyclophorus_expansus

Cyclostoma expansum Pfeiffer, 1853b [1851]: 242. Pfeiffer 1854a: 293, pl. 39, figs 20, 21.
Cyclophorus expansus – Reeve 1861: sp. 18. Hanley and Theobald 1870: 1, pl. 2, figs 3, 4. Kobelt 1902: 129. Kobelt 1908: 656.

#### Type locality.

unknown.

#### Type material.

Lectotype (design. n.), NHMUK 20130086/1 (Fig. 7E; D=30.1 mm, H=25.5 mm, W=5), paralectotypes NHMUK 20130086/2-3 (2 shells; Fig. 7F; D=29.9 mm, H=25.8 mm, W=5; D=27.3 mm, H=23.7 mm, W=5).

#### Remarks.

The original description did not include an illustration or collection locality. Pfeiffer subsequently (1854a: 293, pl. 39, figs 20, 21) re-published the description and figured the species. There are three shells from Cuming’s collection with an original label in Pfeiffer’s handwriting. The specimen which most closely matches with the measurements given in the original description and the illustration in Pfeiffer (1854a) is here designated as the lectotype.

### Cyclophorus fulguratus

(Pfeiffer, 1854)

http://species-id.net/wiki/Cyclophorus_fulguratus

Cyclostoma (Cyclophorus) fulguratum Pfeiffer, 1854b [1852: 63]. Pfeiffer 1854a: 345, pl. 45, figs 9, 10.
Cyclophorus fulguratus – Reeve 1861: sp. 35. Kobelt 1902: 112.

#### Type locality.

unknown

#### Type material.

Lectotype (design. n.), NHMUK 20130117/1 (Fig. 8A; D=28.6 mm, H=25.4 mm, W=5), paralectotypes NHMUK 20130117/2-3 (2 shells; Fig. 8B; D=27.4 mm, H=25.1 mm, W=5; D=29.7 mm, H=25.7 mm, W=5).

#### Remarks.

The original description by Pfeiffer did not give an illustration of the species or a collection locality. Pfeiffer (1854a: 354, pl. 45, figs 9, 10) re-published the description and figured the species. Three shells from Cuming’s collection have an original Pfeiffer label giving the species name and a collection locality of “Arva”, that could perhaps be in Pfeiffer’s handwriting, although a later label states: “Arva’’ added to label not by Pfeiffer? In addition there is a separate label with the specimens, also possibly in Pfeiffer’s handwriting, stating: ‘Prame Pegu’. The figured shell from Pfeiffer (1854a: pl. 45, figs 9, 10) with an “x” written in the aperture is here designated as the lectotype but the type locality remains uncertain.

### Cyclophorus fultoni

Godwin-Austen & Beddome, 1894

http://species-id.net/wiki/Cyclophorus_fultoni

Cyclophorus fultoni Godwin-Austen & Beddome, 1894: 508. Kobelt 1902: 129.

#### Type locality.

Khasi Hills [Meghalaya, India].

#### Type material.

Lectotype (design. n.), NHMUK 1894.6.20.1 (Fig. 8C; D=49.2 mm, H=32.8 mm, W=5).

#### Remarks.

Godwin-Austen stated that he received three specimens from Mr. Fulton. Only one specimen from Hugh Fulton could be located in the NHM collections. The specimen has an original Godwin-Austen label stating “Type” and the shell closely matches with the measurements given in the original description. It is here designated as the lectotype.

### Cyclophorus fuscicolor

Godwin-Austen, 1876

http://species-id.net/wiki/Cyclophorus_fuscicolor

Cyclophorus fuscicolor Godwin-Austen, 1876: 173, pl. 8A, fig. 1. Kobelt 1902: 112.

#### Type locality.

Dafla Hills [Arunachal Pradesh and Assam, India].

#### Type material.

lectotype (design. n.), NHMUK 1903.7.1.1452/1 (Fig. 9A; D=57.5 mm, H=44.8 mm, W=6), paralectotype NHMUK 1903.7.1.1452/2 (1 shell; Fig. 9B; D=50.1 mm, H=40.5 mm, W=6).

#### Remarks.

The original description stated “in some specimens”, which implied that this taxon was based on more than one specimen. There are two specimens from the Godwin-Austen collection with “Type” written on the original label. The specimen figured in the original description is here designated as the lectotype.

### Cyclophorus haughtoni

Theobald, 1858

http://species-id.net/wiki/Cyclophorus_haughtoni

Cyclophorus haughtoni Theobald, 1858: 246. Hanley and Theobald 1870: 1, pl. 1, fig. 3, pl. 3 fig. 6 and pl. 48, fig. 6. Kobelt 1902: 129. Kobelt 1908: 661.

#### Type locality.

Maulmein [Mawlamyine, Myanmar].

#### Type material.

Lectotype (design. n.), NHMUK 1888.12.4.1953 (Fig. 10A; D=42.0 mm, H=36.9 mm, W=5), paralectotype NHMUK 1888.12.4.1954 (1 shell; Fig. 10B; D=41.8 mm, H=32.4 mm, W=5).

#### Remarks.

This species was based on more than one specimen, from Theobald’s collection but only one set of measurement was given. The original description did not include an illustration but subsequently, Hanley and Theobald (1870) figured three illustrations of this species. There are two shells in the NHM collections purchased from W. Theobald, with an original label stating “typical”, and with the collection locality “Moulmein”. The specimen that most closely matches the measurements given in the original description, and the illustration in Theobald (1870: pl. 1, fig. 3 and pl. 48, fig. 6) is designated as the lectotype.

### Cyclophorus himalayanus

(Pfeiffer, 1853)

http://species-id.net/wiki/Cyclophorus_himalayanus

Cyclostoma himalayanum Pfeiffer, 1853b [1851]: 242. Pfeiffer 1853a: 247, pl. 33, figs 10, 11.
Cyclophorus himalayanus – Kobelt 1902: 112. Kobelt 1908: 674.

#### Type locality.

Himalayâ [Himalaya, India].

#### Type material.

Lectotype (design. n.), NHMUK 20130118 (Fig. 10C; D=48.0 mm, H=41.1 mm, W=5).

#### Remarks.

This species was described from specimens in the Cuming collection and only one set of shell measurements was given in the original description. Pfeiffer (1853a: 247, pl. 33, figs 10, 11) republished the description and figured a shell from Cuming’s collection. There is a single shell in the NHM collections from the Cuming collection with an original label stating “the type” which exactly matches Pfeiffer illustration (1853a) and is here designated as the lectotype.

### Cyclophorus ibyatensis

(Pfeiffer, 1854)

http://species-id.net/wiki/Cyclophorus_ibyatensis

Cyclostoma (Cyclophorus) ibyatense Pfeiffer, 1854b [1852]: 62. Pfeiffer 1854a: 349, pl. 45, figs 19, 20.
Cyclophorus ibyatensis – Reeve 1861: sp. 48. Kobelt 1902: 139.

#### Type locality.

Insula Ibyat “Bashee group” [Itbayat Island, Batanes Islands, Philippines].

#### Type material.

Lectotype (design. n.), NHMUK 20130081/1 (Fig. 11A; D=23.0 mm, H=17.7 mm, W=5), paralectotype NHMUK 20130081/2 (1 shell; Fig. 11B; D=22.0 mm, H=17.9 mm, W=5).

#### Remarks.

This species was described from specimens in the Cuming collection and only one set of shell measurements was given in the original description. Pfeiffer (1854a: 349, pl. 45, figs 19, 20) republished the description and figured a shell from Cuming’s collection. There are two shells in the NHM collections with an original label in Pfeiffer’s handwriting giving the species name and original collection locality. The specimen which matches the illustration in Pfeiffer (1854a) and the dimensions given in the original description is here designated as the lectotype.

### Cyclophorus implicatus

Bavay & Dautzenberg, 1908

http://species-id.net/wiki/Cyclophorus_implicatus

Cyclophorus implicatus Bavay & Dautzenberg, 1908: 249. Bavay and Dautzenberg 1909: 285, 286, pl. 9, figs 5–7.

#### Type locality.

Muong Bo, Binh-Lu [Vietnam].

#### Type material.

Paralectotype NHMUK 20130087 from Muong-Bo (Fig. 11C; D=36.5 mm, H=26.4 mm, W=5).

#### Remarks.

The original description does not include an illustration but later, Bavay and Dautzenberg (1909: 285, 286, pl. 9, figs 5–7) republished the description and included illustrations of the species. Fischer-Piette (1950: 176) wrote the “holotype, 37 mm” which we consider to be an inadvertent lectotype designation (ICZN 1999: Art. 74.5). The lectotype is housed in the Muséum National ďHistoire Naturelle, Paris. The NHM specimen from the R.B. Lucas collection (purchased from Dautzenberg) has an original label stating “Type” and giving the collection locality “Muong-Bo” and is considered to be a paralectotype. Further paralectotypes are housed in the Royal Belgian Institute of Natural Sciences, Brussels.

### Cyclophorus kinabaluensis

Smith, 1895

http://species-id.net/wiki/Cyclophorus_kinabaluensis

Cyclophorus kinabaluensis Smith, 1895: 495, pl. 38, fig. 4. Kobelt 1902: 130.

#### Type locality.

Kina Balu, N. Borneo

#### Type material.

Lectotype (design. n.), NHMUK 1894.7.20.38 (Fig. 11D; D=45.1 mm, H=31.1 mm, W=4½), paralectotype NHMUK 1893.6.8.31 (1 shell; Fig. 11E; D=43.7 mm, H=27.3 mm, W=4½).

#### Remarks.

There are two shells in the NHM collections with Smith’s handwriting on the original label. One specimen has a small label with “Type” written on it attached inside the aperture. This specimen corresponds to the figured specimen and the measurements given in the original description and is here designated as the lectotype.

### Cyclophorus koboensis

Godwin-Austen, 1915

http://species-id.net/wiki/Cyclophorus_koboensis

Cyclophorus (Glossostyltis) koboensis Godwin-Austen 1915: 495, pl. 38, fig. 4. Gude 1921: 64.

#### Type locality.

Abor Hills, Kobo, on right bank of Tsanspu or Brahmaputra.

#### Type material.

Lectotype (design. n.), NHMUK 1903.7.1.3579/1 from Kobo, R.B. Brahmaputra, Assam (Fig. 12A; D=30.3 mm, H=21.2 mm, W=5), paralectotypes NHMUK 1903.7.1.3579/2-4 from Kobo, R.B. Brahmaputra, Assam (3 shells; Fig. 12B; D=31.3 mm, H=22.2 mm, W=5; D=32.4 mm, H=22.1 mm, W=5; D=30.6 mm, H=20.7 mm, W=5), NHMUK 1903.7.1.3045 from Ponging, Abor Hills (3 shells; D=33.4 mm, H=21.6 mm, W=5; D=34.1 mm, H=23.5 mm, W=5; D=31.0 mm, H=20.6 mm, W=5), NHMUK 1903.7.1.3117 from Yamney Valley, Abor Hills (2 shells; D=29.5 mm, H=18.6 mm, W=5; D=30.3 mm, H=19.7 mm, W=5).

#### Remarks.

Godwin-Austen’s description was based on five specimen lots with figures of shells from different lots provided in the original description. Three lots were listed as being housed in the NHM, and two lots in the Indian Museum, Calcutta. Each of the three NHM has original labels in Godwin-Austen’s handwriting stating species name, collection locality and catalogue numbers. The figured specimen from the lot 1903.7.1.3579 labelled “cotype” is here designated as the lectotype.

### Cyclophorus labiosus

(Pfeiffer, 1854)

http://species-id.net/wiki/Cyclophorus_labiosus

Cyclostoma (Cyclophorus) labiosum Pfeiffer, 1854d [1853]: 51.
Cyclophorus labiosus – Reeve 1861: sp. 32. Kobelt 1902: 100.

#### Type locality.

Unknown.

#### Type material.

Lectotype (design. n.), NHMUK 20130080 (Fig. 12C, D=42.2 mm, H=30.4 mm, W=5).

#### Remarks.

This species was described from material in the Cuming collection, and the original description does not include an illustration. Later, Reeve (1861: sp. 32) re-described the species and illustrated a shell from the Cuming collection. There is a single shell in the NHM collections from the Cuming collection with an original label in Pfeiffer’s handwriting. This shell is matches the measurements given in the original description and figured in Reeve and is here designated as the lectotype.

### Cyclophorus linguiferus

(Sowerby I, 1843)

http://species-id.net/wiki/Cyclophorus_linguiferus

Cyclostoma linguiferum Sowerby I, 1843a: 31. Sowerby I 1843c: 125, pl. 29, fig. 198. Pfeiffer 1849: 168, pl. 23, figs 1–3.
Cyclophorus linguiferus – Reeve 1861: sp. 23a, b.
Cyclophorus validus var. *linguifera* – Kobelt 1902: 121.

#### Type locality.

Lobock, insulae Bohol [Loboc, Bohol, Philippines].

#### Type material.

Lectotype (design. n.), NHMUK 20110269/1 from Loboc, Bohol Island, Philippines, (Fig. 12D; D=35.4 mm, H=31.5 mm, W=5), paralectotypes NHMUK 20110269/2-3 (2 shells; Fig. 12E; D=33.1 mm, H=26.6 mm, W=5; D=31.1 mm, H=26.9 mm, W=5).

#### Remarks.

The original description included three varieties indicated with “var. a”, “var. b” and “var. c”. Sowerby I subsequently published Thesaurus Conchyliorum (Sowerby I 1843c) with Latin and English descriptions and associated illustrations. Pfeiffer (1849: pl. 23, figs 1–3) and Reeve (1861: sp. 23a, b) published illustrations of the species, however, neither author recognized or used the three varietal forms. These subsequent illustrations are matched with the specimens in the Cuming collection labelled as “var. a”. Therefore we believe this implies that “var. a” is the type series of *Cyclostoma linguiferum* s.s. and the specimens labelled as “var. b” and “var. c” are distinct variants and are therefore excluded from the type series of this nominal species (ICZN 1999: Art. 72.4.1). The specimen of “var. a” illustrated in Sowerby I (1843c: pl. 29, fig. 198), and closest to the dimensions given in the original description is here designated as the lectotype.

Measurements of specimens in the lots previously recognized as “var. b” to “var. c” are given for future reference:

“var. b.” NHMUK 20110270 from Loboc, Bohol Island [Philippines] (3 shells; D=32.6 mm, H=29.3 mm, W=5; D=32.1 mm, H=28.0 mm, W=5; D=30.1 mm, H=25.4 mm, W=5).

“var. c” NHMUK 20110271 from Loboc, Bohol Island [Philippines] (1 shell; D=30.3 mm, H=27.3 mm, W=5).

### Cyclophorus lingulatus

(Sowerby I, 1843)

http://species-id.net/wiki/Cyclophorus_lingulatus

Cyclostoma lingulatum Sowerby I, 1843b: 64. Sowerby I 1843c: 126, pl. 29, figs 208–210. Pfeiffer 1849: 168, pl. 26, figs 6–10.
Cyclophorus lingulatus – Reeve 1861: sp. 49. Kobelt 1902: 114. Kobelt 1907: 573.

#### Type locality.

Island of Siquijod [Siquijor, Philippines]; Deleguete, Zebu Island [Cebu Island, Philippines]; Sibonga, Zebu Island [Cebu Island, Philippines]; Loboc, Bohol Island [Loboc, Bohol, Philippines]; Argao, Zebu Island [Argao, Cebu Island, Philippines].

#### Type material.

Lectotype (design. n.), NHMUK 20110272/1 from island of Siquijod (Fig. 12F; D=21.3 mm, H=16.9 mm, W=4½), paralectotypes NHMUK 20110272/2-3 (2 shells; Fig. 12G; D=20.9 mm, H=17.5 mm, W=4½; D=20.0 mm, H=17.2 mm, W=4½).

#### Remarks.

The original description included eight varieties indicated as “var. a” to “var. h.”, based on samples from various localities sampled by H. Cuming and cites an illustration in “Thesaurus Conchyliorum part 3, pl. 30, fig. 208”. This illustration matches the specimens in Cuming collection labelled as “var. a”. Therefore we believe this implies that “var. a” is the type series of *Cyclostoma linguiferum* s.s. and the specimens labelled as “var. b” to “var. h” are distinct variants and are therefore excluded from the type series of this nominal species (ICZN 1999: Art. 72.4.1). The specimen of “var. a” illustrated by Sowerby I (1843c: pl. 29, fig. 208) is here designated as the lectotype.

Measurements of specimens in the lots previously recognized as “var. b” to “var. h” are given here for future reference:

“var. b.” NHMUK 20110273 from Siquijod Island [Philippines] (3 shells; D=19.9 mm, H=15.8 mm, W=4½; D=20.6 mm, H=16.5 mm, W=4½; D=20.4 mm, H=16.1 mm, W=4½).

“var. c” NHMUK 20110274 from Deleguete, Zebu Island [Philippines] (3 shells; D=26.0 mm, H=20.9 mm, W=4½; D=23.6 mm, H=18.4 mm, W=4½; D=23.5 mm, H=18.5 mm, W=4½).

“var. d” NHMUK 20110275 from Deleguete, Zebu Island [Philippines] (3 shells; D=24.1 mm, H=18.6 mm, W=4½; D=24.5 mm, H=19.9 mm, W=4½; D=24.5 mm, H=18.2 mm, W=4½).

“var. e” NHMUK 20110276 from Sibonga, Zebu Island [Philippines] (3 shells; D=24.4 mm, H=19.3 mm, W=4 ½; D=22.4 mm, H=19.8 mm, W=4 ½; D=24.4 mm, H=19.6 mm, W=4 ½).

“var. f” NHMUK 20110277 from Loboc, Bohol Island [Philippines] (3 shells; D=25.6 mm, H=20.1 mm, W=4½; D=25.4 mm, H=20.4 mm, W=4½; D=24.9 mm, H=19.3 mm, W=4½).

“var. g” NHMUK 20110278 from Argao, Zebu Island [Philippines] (3 shells; D=24.4 mm, H=18.6 mm, W=4½; D=23.6 mm, H=19.8 mm, W=4½; D=23.4 mm, H=19.4 mm, W=4½).

“var. h” NHMUK 20110279 from Loboc, Bohol Island [Philippines] (3 shells; D=25.1 mm, H=19.6 mm, W=4½; D=25.6 mm, H=20.6 mm, W=4½; D=26.4 mm, H=20.9 mm, W=4½).

### Cyclophorus malayanus

(Benson, 1852)

http://species-id.net/wiki/Cyclophorus_malayanus

Cyclostoma malayanum Benson, 1852: 269.
Cyclophorus malayanus – Reeve 1861: sp. 2. Kobelt 1902: 130. Kobelt 1908: 658.

#### Type locality.

In montibus vallibusque Insularum Penang et Lancavi, necnon in Peninsula Malayana [In the mountains, the valleys of the islands of Penang and Langkawi, as well as the Peninsula Malaysia]

#### Type material.

Syntypes NHMUK 20130089 (2 shells; Fig. 13A; D=43.5 mm, H=32.1 mm, W=5; D=47.0 mm, H=37.3 mm, W=5)

#### Remarks.

The original description did not include an illustration but Reeve (1861: sp. 2) subsequently re-published the description with illustrations of a specimen from the Cuming collection. The NHM collection contains a lot of three specimens’ from the Cuming collection labelled “Malay Peninsula”. A label reads “Mr. Benson has also sent me his *Malayanum* and the true *volvulus* for comparison….”. Two of the three shells are close to the measurements and description in the original description and the label, presumably written by Cuming, indicates that these are the three specimens sent to Cuming by Benson, of which two without opercula are syntypes. The third specimen with an operculum being what Benson considered to be ‘*volvulus*’ and not a member of the type series of ‘*malayanus*’. There is a further lot from “Pulo Penang” housed in the University Museum of Zoology Cambridge with original Benson labels including one specimen labelled by Benson as ‘Type’.

### Cyclophorus monachus

(Morelet, 1866)

http://species-id.net/wiki/Cyclophorus_monachus

Cyclostoma monachus Morelet, 1866: 166.
Cyclophorus monachus – Kobelt 1902: 100. Kobelt 1908: 619.

#### Type locality.

Cochinchina [Saigon, Vietnam].

#### Type material.

Lectotype (design. n.), NHMUK 1893.2.4.499 (Fig. 13B; D=38.2 mm, H=23.7 mm, W=5), paralectotype NHMUK 1893.2.4.500 (1 shell; Fig. 13C; D=35.2 mm, H=22.1 mm, W=5).

#### Remarks.

The original description did not include an illustration and only one set of shell measurements was given. There are two specimens in the NHM collections with an original label stating “Type” and giving the reference of the original description. The shell that most closely matches with the measurement in the original description and with an “x” written in the aperture is here designated as the lectotype.

### Cyclophorus muspratti

Godwin-Austen & Beddome, 1894

http://species-id.net/wiki/Cyclophorus_muspratti

Cyclophorus muspratti Godwin-Austen & Beddome, 1894: 506. Kobelt 1902: 101. Kobelt 1908: 662.

#### Type locality.

Naga Hills, and Maokokehung, Naga Hills [Assam, Arunachal Pradesh and Nagaland, India].

#### Type material.

Holotype NHMUK 1903.7.1.1427/1 from Naga Hills (Fig. 14A; D=48.7 mm, H=36.4 mm, W=5), paratypes NHMUK 1903.7.1.1427/2-4 (3 shells: 2 adults and 1 juvenile; Fig. 14B; D=50.2 mm, H=38.1 mm, W=5; D=47.7 mm, H=38.1 mm, W=5).

#### Remarks.

The original description included two sets of shell measurements (the type and the largest specimen), and Godwin-Austen explicitly stated there to be a unique name-bearing type. The NHM collections contain a lot of four shells from the Godwin-Austen collection (ex. Doherty collection) and have his original handwritten label stating “Type”. The specimen with ‘type’ written on the shell most closely matches with the ‘type’ shell dimensions given in the original description and is here considered to be the holotype, the remaining three shells being paratypes.

### Cyclophorus nagaensis

Godwin-Austen & Beddome, 1894

http://species-id.net/wiki/Cyclophorus_nagaensis

Cyclophorus nagaensis Godwin-Austen & Beddome, 1894: 507. Kobelt 1902: 101.

#### Type locality.

Naga Hills, near Khonoma and Kigwema, 5000–6000 feet; Maokokehung [Assam, Arunachal Pradesh and Nagaland, India].

#### Type material.

Lectotype (design. n.), NHMUK 1903.7.1.1456/1 from Naga Hills (Fig. 15A; D=45.2 mm, H=35.5 mm, W=5), paralectotypes NHMUK 1903.7.1.1456/2-4 (3 shells; Fig. 15B; D=43.9 mm, H=34.1 mm, W=5; D=44.7 mm, H=35.7 mm, W=5; D=42.3 mm, H=32.7 mm, W=5).

#### Remarks.

The NHM collections contain a lot of four shells from the Godwin-Austen collection (ex. Doherty collection) and have his original handwritten label stating “Type”. The specimen that most closely matches the original description and the measurements given by Godwin-Austen is designated as the lectotype.

### Cyclophorus niahensis

Godwin-Austen, 1889

http://species-id.net/wiki/Cyclophorus_niahensis

Cyclophorus niahensis Godwin-Austen, 1889: 334, pl. 35, fig. 1. Kobelt 1902: 115.

#### Type locality.

Niah Hills [Sarawak, Malaysia].

#### Type material.

Lectotype (design. n.), NHMUK 1889.12.7.3 (Fig. 15C; D=44.0 mm, H=28.0 mm, W=4), paralectotype NHMUK 1889.12.7.4 (2 shells: 1 adult and 1 juvenile; Fig. 15D; D=41.1 mm, H=24.8 mm, W=4).

#### Remarks.

The use of the term “holotype” in Vermeulen (1999: 144) does not constitute a valid lectotype designation, since there was no explicit indication to a particular specimen (ICZN 1999: Art. 74.5). The specimen which most closely matches the figure in the original description (especially in respect to the position of interrupted growth lines on last whorl) and marked with an “X” on the inside of the aperture is here designated as the lectotype.

### Cyclophorus cochranei ochraceus

Godwin-Austen, 1889

http://species-id.net/wiki/Cyclophorus_cochranei_ochraceus

Cyclophorus cochranei var. *ochraceus* Godwin-Austen, 1889: 334, 335. Kobelt 1902: 127.

#### Type locality.

Busan Hills [Sarawak, Malaysia].

#### Type material.

Lectotype NHMUK 1889.12.7.6 (Fig. 16A; D=41.7 mm, H=27.4 mm, W=5).

#### Remarks.

The original description clearly stated the taxon was based on two specimens from Busan Hills, and the unique name-bearing type was not stated. However, there is only one remaining specimen from the Godwin-Austen type lot in the NHM collections. Subsequent use of the term “holotype” in Vermeulen (1999: 144) is seemed an unambiguously selected a particular specimen as the name-bearing type. This constitutes a valid lectotype designation (ICZN 1999: Art. 74.5).

### Cyclophorus phlegethon

Godwin-Austen, 1889

http://species-id.net/wiki/Cyclophorus_phlegethon

Cyclophorus phlegethon Godwin-Austen, 1889: 335, 336. Kobelt 1902: 131.

#### Type locality.

Molu Hills [Sarawak, Malaysia].

#### Type material.

Holotype NHMUK 1998011 (Fig. 16B; D=39.1 mm, H=23.3 mm, W=4).

#### Remarks.

Godwin-Austen clearly stated that this taxon was described based on only one specimen, therefore we recognise this specimen as the holotype fixed by monotypy (ICZN 1999: Art. 73.1.2).

### Cyclophorus picturatus

(Pfeiffer, 1854)

http://species-id.net/wiki/Cyclophorus_picturatus

Cyclostoma (Cyclophorus) picturatum Pfeiffer, 1854b [1852]: 62. Pfeiffer 1854a: 347, pl. 45, figs 13, 14.
Cyclophorus picturatus – Reeve 1861: sp. 22. Kobelt 1902: 116. Kobelt 1907: 596.

#### Type locality.

Unknown.

#### Type material.

Lectotype (design. n.) NHMUK 20130082/1 (Fig. 16C, D; D=29.1 mm, H=22.5 mm, W=4½), paralectotypes NHMUK 20130082/2-3 (2 shells; Fig. 16D, D=30.0 mm, H=23.1 mm, W=4½; D=30.4 mm, H=20.8 mm, W=4½).

#### Remarks.

This species was described based on specimens from the Cuming collection. Pfeiffer (1854a: pl. 45, figs 13, 14.) re-published the description and figured this species. The NHM collections contain a lot of three shells from the Cuming collection with original labels in Pfeiffer’s handwriting stating the species name. None of these shells exactly match with the illustration in Pfeiffer (1854a). However, the specimen mostly similar to the illustration in Reeve (1861: sp. 22) is illustrated here and is designated as the lectotype.

### Cyclophorus poeciloneurus

Godwin-Austen & Beddome, 1894

http://species-id.net/wiki/Cyclophorus_poeciloneurus

Cyclophorus poeciloneurus Godwin-Austen & Beddome, 1894: 507, 508. Kobelt 1902: 102. Kobelt 1908: 639.

#### Type locality.

Lahúpa Naga Hills, Munipur, and eastward to the Dihing [India].

#### Type material.

Lectotype (design. n.), NHMUK 1903.7.1.1522/1 from Lahúpa Naga Hills (Fig. 16E; D=31.1 mm, H=24.9 mm, W=5), paralectotype NHMUK 1903.7.1.1522/2 (1 shell; Fig. 16F; D=27.3 mm, H=20.2 mm, W=5).

#### Remarks.

The authors indicated that four lots of specimens were examined in the original description (from the Godwin-Austen, Ogel, Doherty and Beddome collections). In addition, the authors stated “Type” in relation to the specimens from the Godwin-Austen collection, which consist of two shells. The original description did not include an illustration, and only one set of shell measurement was given. The specimen that has a small label stating “Type” glued inside the aperture, and which matched the measurements given in original description is here designated as the lectotype. The paralectotypes from the Ogel, Doherty, and Beddome collections were not found.

### Cyclophorus fulguratus rangunensis

Kobelt, 1908

http://species-id.net/wiki/Cyclophorus_fulguratus_rangunensis

Cyclophorus fulguratus var. Pfeiffer, 1869: 440, pl. 98, figs 1, 2.
Cyclophorus fulguratus var. *rangunensis* Kobelt, 1908: 647, pl. 93, figs 1, 2. Gude 1921: 61.

#### Type locality.

Inter Thyet-Mio et Rangoon Birmanorum, Pegu [between Thayet District and Yangon in Myanmar, Bago].

#### Type material.

Lectotype (design. n.), NHMUK 20130091/1 (Fig. 17A; D=34.7 mm, H=30.3 mm, W=5), paralectotypes NHMUK 20130091/2-3 (2 shells; Fig. 17B; D=27.4 mm, H=23.4 mm, W=5; D=27.1 mm, H=23.2 mm, W=5).

#### Remarks.

Kobelt (1908) described this species based on Pfeiffer’s specimens. Since a range of measurement was given it can be assumed that the taxon was described using more than one specimen. There are three shells in the NHM collections from the Cuming collection with original labels in Pfeiffer’s handwriting. One of the specimens matches with figures in Kobelt (1908: pl. 93, figs. 1, 2) and Pfeiffer (1869: pl. 98, figs. 1, 2) and is here designated as the lectotype.

### Cyclophorus eximus rouyeri

Bullen, 1906

http://species-id.net/wiki/Cyclophorus_eximus_rouyeri

Cyclophorus eximus var. *rouyeri* Bullen, 1906: 343, pl. 25, fig. 5. Kobelt 1908: 680.

#### Type locality.

Mount Singalong [Mount Singgalang, West Sumatra, Indonesia].

#### Type material.

Lectotype (design. n.), NHMUK 1906.1.16.51 (Fig. 17C; D=50.1 mm, H=40.5 mm, W=5), paralectotype NHMUK 20130078 (1 shell; Fig. 17D; D=50.1 mm, H=40.5 mm, W=5).

#### Remarks.

The original description was clearly based on more than one specimen, but only one set of measurements and illustrations were given. There are two shells, from two lots in the NHM collections which are both considered to be part of the original type series. The specimen figured in the original description is here designated as the lectotype

### Cyclophorus saturnus

Pfeiffer, 1862

http://species-id.net/wiki/Cyclophorus_saturnus

Cyclophorus saturnus Pfeiffer, 1862: 116, pl. 12, fig. 6. Kobelt 1902: 132.

#### Type locality.

Camboja [Cambodia].

#### Type material.

Lectotype (design. n.), NHMUK 20130119/1 (Fig. 18A; D=63.1 mm, H=46.5 mm, W=5½), paralectotypes NHMUK 20130119/2-3 (2 shells; Fig. 18B; D=57.7 mm, H=43.2 mm, W=5½; D=60.0 mm, H=46.8 mm, W=5½).

#### Remarks.

This species was described based on a specimen collected by M. Mouhot from the Cuming collection, and only one set of shell measurements and one specimen was illustrated in the original description. There are three shells in the NHM collections from the Cuming collection with an original label in Pfeiffer’s handwriting stating the taxon name, collector and collection locality. The figured specimen with an “x” written inside the aperture is here designated as the lectotype. The type locality of Cambodia applied to contemporaneous boundaries but Mouhot also collected in an area that now comes within the boundary of southern Vietnam.

### Cyclophorus schepmani

Laidlaw, 1957

http://species-id.net/wiki/Cyclophorus_schepmani

Cyclophorus schepmani Laidlaw, 1957: 126, 127.

#### Type locality.

Sinabang, Simalur Island, West Sumatra.

#### Type material.

Paratype NHMUK 1957.11.18.7 (1 shell; Fig. 19A; D=44.4 mm, H=35.7 mm, W=5 ½).

#### Remarks.

The authors indicated that four lots of specimens were examined in the original description. The original description did not include an illustration, and two set of shell measurement were given. However, the holotype was clearly designated and is housed in the Leiden Museum, Netherlands (now Naturalis Biodiversity Centre). The NHM registration records show that this specimen was purchased from Laidlaw, ex. Dr. Jacobson collection, and the original label states ‘paratype’. The locality given by Laidlaw was “2 ex. July; Sinabang” match with the specimen. We therefore consider the single specimen as paratype.

### Cyclophorus serratizona

Hanley & Theobald, 1876

http://species-id.net/wiki/Cyclophorus_serratizona

Cyclophorus serratizona Hanley & Theobald, 1876: 57, pl. 144, fig. 7. Kobelt 1908: 654. Gude 1921: 77.

#### Type locality.

Upper Salwen [Myanmar].

#### Type material.

Possible syntypes NHMUK 88.12.4.1955 (3 shells, Fig. 19B; D=37.1 mm, H=30.0 mm, W=5; D=39.9 mm, H=30.8 mm, W=5; D=40.7 mm, H=32.9 mm, W=5).

#### Remarks.

This taxon was described based on specimens from the Theobald collection. Coan and Kabat (2012: 326) stated that the types could not be located in either the NHM or the Leeds Museum. We located 3 specimens in the NHM general collection with a label stating that they were purchased from Theobald, “type figd in C. I.”, but giving Moulmein (currently Mawlamyine), farm (=from?) caves, as the locality, which is at the mouth of the Salween. It seems likely that there is a mix up in the documentation of the locality but we cannot determine if the error is in the locality given in the original description, an error in the labelling with the specimens or if this lot is simply not type material. We therefore treat the material as possible syntypes.

The locality given by Hanley and Theobald was Upper Salwen (Salween or currently Thanlwin). However, the material identified as the syntype series carries labels giving Moulmein (currently Mawlamyine), farm (=from?) caves, as the locality, which is at the mouth of the Salween. As the Salween is close to 3,000 km in length and the river originates from the Tibetan Plateau, it is clear that ‘Upper Salwen’ is inaccurate. It is conceivable that the intended record was Upper Myanmar but on current evidence we conclude that the Mawlamyine is the type locality.

### Cyclophorus siamensis

(Sowerby I, 1850)

http://species-id.net/wiki/Cyclophorus_siamensis

Cyclostoma siamense Sowerby I, 1850: 158, pl. 31a, figs 292, 293. Pfeiffer 1854a: 323, pl. 42, figs 5, 6.
Cyclophorus siamensis – Reeve 1861: sp. 19. Kobelt 1902: 132.
Cyclophorus khasiensis Nevill, 1878: 273 (‘new replacement name’).

#### Type locality.

Siam [Thailand].

#### Type material.

lLectotype (design. n.), NHMUK 20130088/1 (Fig. 20A; D=51.2 mm, H=40.0 mm, W=5), paralectotype NHMUK 20130088/2 (1 shell; Fig. 20B; D=49.8 mm, H=39.5 mm, W=5).

#### Remarks.

The original description as well as those in Pfeiffer (1854a: 323) are particularly accurate, both showing the dark banding pattern and varix on the last whorl, and both figures appear to be from the same specimen. The NHM collections contain two shells from the Cuming collection with original labels stated the taxon name, type locality and “f. 292, 293”. The specimen which corresponds to the illustrations in Sowerby I (1850) and Pfeiffer (1854a) is here designated as the lectotype.

Nevill (1878: 273) stated that *Cyclostoma siamensis* Sowerby I, 1850 occur in Khasi Hills, India not in Siam. Nevill considered this is an inappropriate taxon name, and *Cyclophorus khasiensis* Nevill, 1878 was nominated as a new replacement name based on specimens from the Godwin-Austen collection. This is however an unjustified replacement name, and therefore a junior objective synonym of *Cyclostoma siamensis* Sowerby I, 1850 (ICZN 1999: Arts 18, 72.7). The specimens of *Cyclophorus khasiensis* in the Godwin-Austen collection have no nomenclatural status.

It should be noted that Kobelt (1902) attributed the date of publication of this species in error as “1843”. For the correct dates of publication for “Thesaurus Conchyliorum” see Petit (2009: 32).

### Cyclophorus spironema

(Pfeiffer, 1855)

http://species-id.net/wiki/Cyclophorus_spironema

Cyclostoma (Cyclophorus) spironema Pfeiffer, 1855a [1854]: 127.
Cyclophorus spironema – Kobelt 1902: 104.

#### Type locality.

India.

#### Type material.

Lectotypes (design. n.), NHMUK 20130083/1 (Fig. 21A; D=27.9 mm, H=19.8 mm, W=4), paralectotypes NHMUK 20130083/2-3 (2 shells; Fig. 21B; D=26.8 mm, H=21.4 mm, W=4; D=28.2 mm, H=20.9 mm, W=4).

#### Remarks.

This species was described based on specimens from the Cuming collection. The NHM collections contain three shells from the Cuming collection with an original label in Pfeiffer’s handwriting giving the taxon name and collection locality. The specimen figured in Gude (1921: 55, 56, fig. 13) does not constitute a lectotype designation; as Gude did not select a particular syntype to be the unique name-bearing type (ICZN 1999: Art. 74.3). We here designate the specimen figured in Gude (1921: fig. 13) as the lectotype.

### Cyclophorus subblaevigatus

Blanford, 1869

http://species-id.net/wiki/Cyclophorus_subblaevigatus

Cyclophorus subblaevigatus Blanford, 1869: 446, 447. Hanley and Theobald 1870: 16, pl. 34, fig. 7. Kobelt 1902: 133.

#### Type locality.

haud procul a Bhamo, ad ripas fluminis Iravadi [not far from the Bhamo, on the banks of the Iravadi River, Myanmar].

#### Type material.

Lectotype (design. n.), NHMUK 196550 (Fig. 21C; D=46.1 mm, H=30.8 mm, W=5).

#### Remarks.

The term “nonnunquam” (“sometimes”) in the original description of shell shape appears to imply that this taxon was based on more than one specimen although only one set of measurements was given in the original description. The use of “the type” in Hanley and Theobald (1870: 16) may not to constitute a valid lectotype designation because a label stating that it is the figured specimen is not in Hanley or Theobald’s hand and it is not clear if only one specimen was available to Hanley and Theobald. We therefore treat “the type” attribution as an invalid, inadvertent lectotype designation (ICZN 1999: Art. 74.5). The single specimen in the NHM from the Blanford collection and figured in Hanley and Theobald (1870: pl. 34, fig. 7), is here designated as the lectotype.

### Cyclophorus taeniatus

(Pfeiffer, 1855)

http://species-id.net/wiki/Cyclophorus_taeniatus

Cyclostoma (Cyclophorus) taeniatum Pfeiffer, 1855b [1854]: 301.
Cyclophorus taeniatus – Reeve 1861: sp. 39. Kobelt 1902: 134.

#### Type locality.

Sumatra.

#### Type material.

Lectotype (design. n.), NHMUK 20130120 (Fig. 21D; D=28.1 mm, H=23.6 mm, W=5).

#### Remarks.

This species was described based on specimens from the Cuming collection. The NHM collections contain a single specimen from the Cuming collection with an original label in Pfeiffer’s handwriting giving the taxon name and collection locality. This single specimen closely matches with the measurements given in the original description and the illustration in Reeve (1861: sp. 39) and is here designated as the lectotype.

### Cyclophorus talboti

Godwin-Austen, 1889

http://species-id.net/wiki/Cyclophorus_talboti

Cyclophorus talboti Godwin-Austen, 1889: 335. Kobelt 1902: 119.

#### Type locality.

Busan Hills [Sarawak, Malaysia].

#### Type material.

Lectotype, NHMUK 1889.12.7.7 (Fig. 21E; D=40.1 mm, H=27.5 mm, W=5)

#### Remarks.

Godwin-Austen stated that this taxon was named after Captain Talbot and based on specimens from the collection of A. Everett. The NHM collections contain a lot of three specimens mounted on a single specimen board. The specimen labelled 1889.12.7.7 is marked with the word type, and the original description details. The two other specimens are from the W. Jeakes Esq. (NHMUK 1859.3.30.12) and C. Hose Esq. (1893.3.10.3) collections and have no associated locality data and are therefore excluded from the type series. The use of the term “holotype” in Vermeulen (1999: 144) is an inadvertent lectotype designation since a particular shell was selected to be the unique name-bearing type (ICZN 1999: Art. 74.5).

### Cyclophorus tigrinus

(Sowerby I, 1843)

http://species-id.net/wiki/Cyclophorus_tigrinus

Cyclostoma tigrinum Sowerby I, 1843a: 30. Sowerby I 1843c: 126, pl. 29, figs 201–204. Pfeiffer 1848: 61, pl. 8, figs 13–16.
Cyclophorus tigrinus – Kobelt 1907: 578.

#### Type locality.

Unknown.

#### Type material.

Lectotype (design. n.), NHMUK 20110231/1 (Fig. 22A; D=32.1 mm, H=28.2 mm, W=6), paralectotypes NHMUK 20110231/2-3 (2 shells; Fig. 22B; D=28.0 mm, H=25.5 mm, W=6; D=30.4 mm, H=26.2 mm, W=6).

#### Remarks.

The original description of this species included seven varieties indicated with “var. a” to “var. g” from the Cuming Collection. Latin and English descriptions associated with illustrations were then published in the “Thesaurus Conchyliorum” (Sowerby I 1843c). None of the subsequent authors recognized or used these seven varietal names. Two further works provided illustrations of the species from the Cuming coll. (Pfeiffer 1848: pl. 8, figs 13–16; pl. 16, figs 17–20; Reeve 1861: sp. 25a, b; 8 fig. 30). These subsequently published illustrations match the specimens in the Cuming collection labelled as “var. a”. Therefore we believe this implies that “var. a” is the type series of *Cyclostoma tigrinum* s.s. and the specimens labelled as “var. b” are distinct variants and are therefore excluded from the type series of this nominal species (ICZN 1999: Art. 72.4.1). The specimen of “var. a” illustrated by Sowerby I (1843c: pl. 29, figs 201, 202) is here designated as the lectotype.

Measurements of specimens in “var. b” are given for future reference:

“var. b.” NHMUK 20110232 from Guimaras Island [Philippines] (3 shells; D=30.5 mm, H=26.5 mm, W=6; D=28.0 mm, H=26.6 mm, W=6; D=27.2 mm, H=22.3 mm, W=6).

### Cyclophorus tuba

(Sowerby I, 1842)

http://species-id.net/wiki/Cyclophorus_tuba

Cyclostoma tuba Sowerby I, 1842: 83. Sowerby I 1843c: 122, pl. 27, figs 129, 130. Pfeiffer 1849: 169, pl. 23, figs 10, 11.
Cyclophorus tuba – Reeve 1861: sp. 9. Kobelt 1902: 134.

#### Type locality.

prope Montem Ophir, Malaccae [Gunung Ledang, Johor, Malaysia].

#### Type material.

Lectotype (design. n.), NHMUK 20120064/1 (Fig. 22C; D=51.1 mm, H=35.7 mm, W=5), paralectotype NHMUK 20120064/2 (1 shell; Fig. 22D; D=48.1 mm, H=33.4 mm, W=5).

#### Remarks.

The original description of this species included two un-named varieties from the Cuming Collection. The NHM collection contain two lots from the Cuming collection with the original labels giving the taxon name, type locality and varietal names stated as “var. a” and “var. b”. Latin and English descriptions associated with illustrations were then published in the “Thesaurus Conchyliorum” (Sowerby I 1843c). Two further works provided illustrations of the species from Cuming coll. (Pfeiffer 1849: 169, pl. 23, figs 10, 11; Reeve 1861: sp. 9). These subsequently published illustrations match specimens in the Cuming collection labelled as “var. a”. We consider that this implies that “var. a” is the type series of *Cyclostoma tuba* s.s. and the specimens labelled as “var. b” are distinct variants and are therefore excluded from the type series (ICZN 1999: Art. 72.4.1). The specimen of “var. a” illustrated by Sowerby I (1843c: 122, pl. 27, figs 129, 130) is here designated as the lectotype.

Measurements of specimens in “var. b” are given for future reference:

“var. b.” NHMUK 20120065 from Mountain Ophir, Malaccae [Malaysia] (3 shells; D=50.4 mm, H=33.8 mm, W=5; D=50.1 mm, H=35.5 mm, W=5; D=48.1 mm, H=34.0 mm, W=5).

### Cyclophorus turgidus

(Pfeiffer, 1851)

http://species-id.net/wiki/Cyclophorus_turgidus

Cyclostoma turgidum Pfeiffer, 1851: 139, 140 (‘new replacement name’). Pfeiffer 1853a: 257, pl. 35, fig. 15, 16.
Cyclostoma crassum Pfeiffer, 1853b [1851: 242] (non C.B. Adams 1851).
Cyclophorus turgidus – Reeve 1861: sp. 43.
Cyclophorus crassus – Kobelt 1902: 136, 137.

#### Type locality.

Liew Kiew [Ryukyu Islands, Japan].

#### Type material.

Lectotype (design. n.), NHMUK 20040591/1 (Fig. 23A; D=27.1 mm, H=23.1 mm, W=5), paralectotypes NHMUK 20040591/2-3 (2 shells; Fig. 23B; D=25.9 mm, H=21.3 mm, W=5; D=25.2 mm, H=20.5 mm, mm, W=5).

#### Remarks.

The name *Cyclostoma turgidum* Pfeiffer, 1851 was presented as a replacement name for *Cyclostoma crassum* Pfeiffer, 1853, a junior homonym. However since the “*crassum*” description was not published until 1853 (in the Proceedings of the Zoological Society for 1851 volume, see Duncan 1937) *Cyclostoma turgidum* is the valid original description. This taxon was described and illustrated based on specimens from the Cuming collection. The NHM collections contain two lots from the Cuming collection that have original labels in Pfeiffer’s handwriting with a striking-through of the taxon name “*crassum*”, replaced with “*turgidum*”. One lot of 3 specimens, NHMUK 20040591, has the collection locality “Liew Kiew” which matches with that given in the original description. The specimen figured in Pfeiffer (1853a: pl. 35, figs 15, 16) which matches with the measurements given in the original description is here designated as the lectotype. The second lot of three shells, NHMUK 20040590, has the collection locality “Ibyat, Bashee Islands” which corresponds to “var. minor in insula Ibyat (Bashee group)” from the Pfeiffer’s (1853b) “*crassum*” description and is excluded from the type series of this nominal species (ICZN 1999: Art. 72.4.1).

Measurements of the specimens “var. minor” are given for future reference:

“var. minor” NHMUK 20040590 from Ibayat, Bashee Island [Batan, Island, Philippines] (3 shells; D=20.5 mm, H=16.8 mm, W=5; D=21.0 mm, H=17.9 mm, W=5; D=20.8 mm, H=16.3 mm, W=5).

### Cyclophorus validus

(Sowerby I, 1842)

http://species-id.net/wiki/Cyclophorus_validus

Cyclostoma validum Sowerby I, 1842: 82. Sowerby I 1843c: 123, pl. 27, figs 132, 133. Pfeiffer 1848: 89, pl. 11, figs 9, 10.
Cyclophorus validus – Reeve 1861: sp. 23c, d. Kobelt 1902: 120. Kobelt 1908: 581.

#### Type locality.

Island of Leyte, island of Luçon, island of Samar and island of Mindanao, Philippines.

#### Type material.

Lectotype (design. n.), NHMUK 20110280/1 from island of Leyte (Fig. 23C; D=47.9 mm, H=40.4 mm, W=5), paralectotypes NHMUK 20110280/2-3 (2 shells; Fig. 23D; D=46.7 mm, H=39.9 mm, W=5; D=44.0 mm, H=37.6 mm, W=5).

#### Remarks.

The original description of this species included four varieties indicated “var. a” to “var. d”, without illustration from the Cuming Collection. Subsequent authors (including Sowerby I 1843b) did not recognise or use these varietal names. The NHM collections contain four lots of Cuming collection material with original labels giving the taxon and varietal names “var. a” to “var. d”. The description and illustration in Sowerby I (1843b) match the specimens in the Cuming collection labelled as “var. a”. We consider that this implies that “var. a” is the type series of *Cyclostoma validum* s.s. and the specimens labelled as “var. b”, “var. c” and “var. d” are distinct variants and are therefore excluded from the type series (ICZN 1999: Art. 72.4.1). The specimen of “var. a” illustrated by Sowerby I (1843c: pl. 27, figs 132, 133), is here designated as the lectotype.

Measurements of specimens in “var. b” to “var. d.” are given for future reference:

“var. b.” NHMUK 20110281 from Tayabas Province, Luzon [Philippines] (3 shells; D=39.2 mm, H=35.8 mm, W=5; D=38.2 mm, H=33.9 mm, W=5; D=36.8 mm, H=34.2 mm, mm, W=5).

“var. c.” NHMUK 20110282 from Catbalonga and Basay, Samar Island [Philippines] (3 shells; D=43.5 mm, H=34.8 mm, W=5; D=43.8 mm, H=34.6 mm, W=5; D=44.5 mm, H=34.9 mm, W=5).

“var. d.” NHMUK 20110283 from Cagayan, Misamis Province, Mindanao Island, Luzon [Philippines] (3 shells; D=35.6 mm, H=28.4 mm, W=5; D=33.1 mm, H=26.1 mm, W=5; D=32.7 mm, H=25.4 mm, mm, W=5).

## Plates

**Figure 1.**
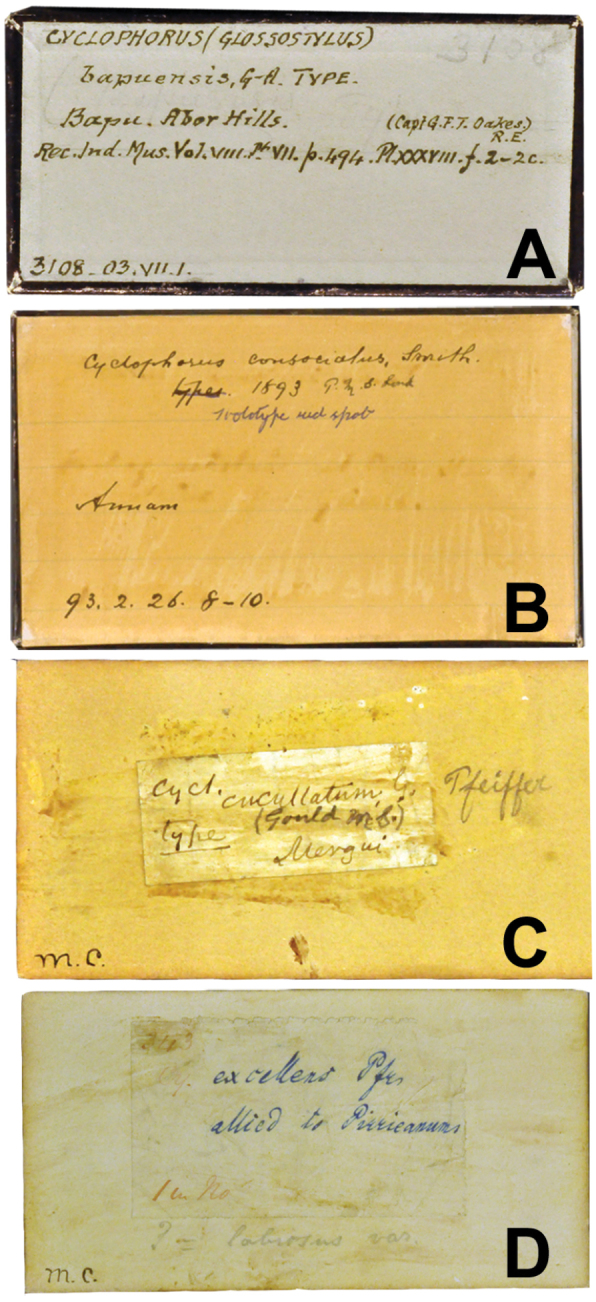
**A** Original labels of *Cyclophorus bapuensis* bearing the author’s, H.H. Godwin-Austen handwriting **B** Original labels of *Cyclophorus consociatus* bearing the author’s, E.A. Smith handwriting. Note that the strikethrough on the “type” and “Holotype red spot” with blue pen are possibly added later by the NHM assistant **C** The original label of *Cyclophorus cucullatus* marked with “Type” is not frequently occurred in Cuming collection, which the possibly indicates specimen received from A.A. Gould **D** The small glued-label written with blue ink on “*excellens* Pfr” and “allied to *pirrieanum*” are Pfeiffer’s handwritten.

**Figure 2.**
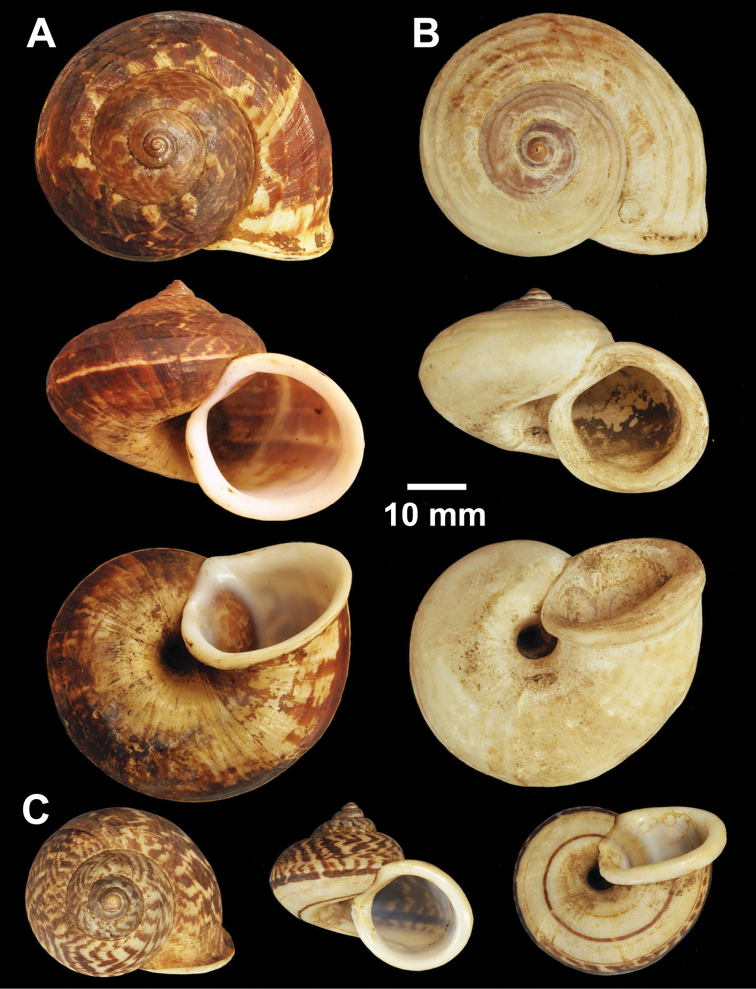
Types of *Cyclophorus* species. **A, B** *Cyclophorus aborensis* Godwin-Austen, 1915 **A** lectotype NHMUK 1903.7.1.3051 and **B** paralectotype NHMUK 1903.7.1.3048 **C** *Cyclophorus affinis* Theobald, 1858, lectotype NHMUK 1903.7.1.1454.

**Figure 3.**
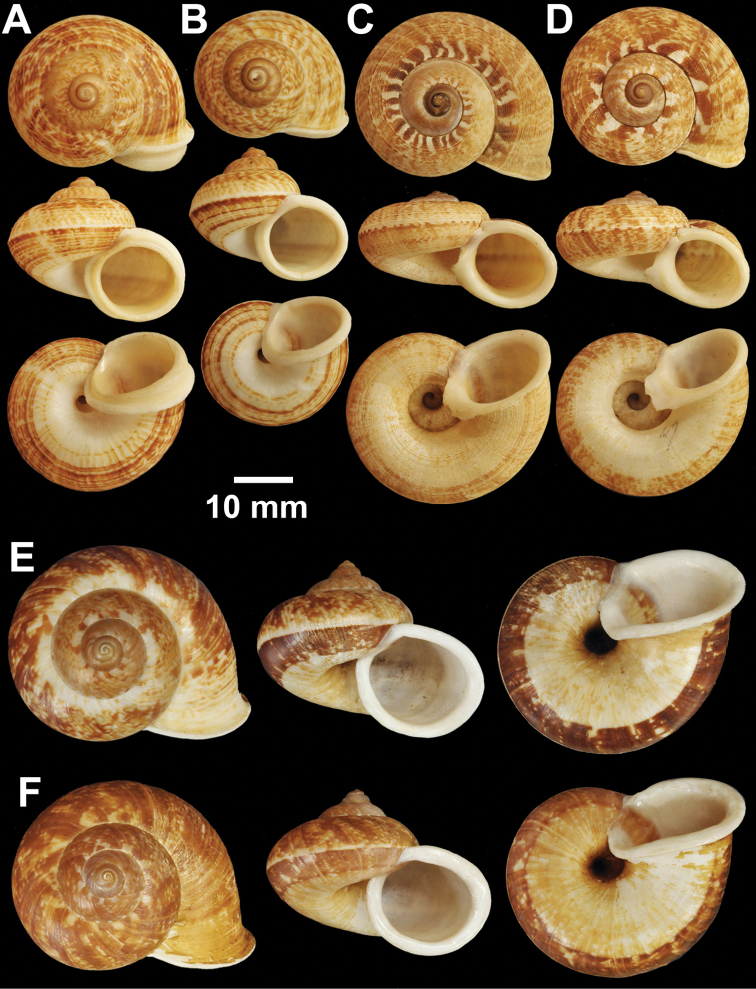
Types of *Cyclophorus* species. **A, B** *Cyclophorus amoenus* (Pfeiffer, 1854) **A** lectotype NHMUK 20130113/1, and **B** paralectotype NHMUK 20130113/2 **C, D** *Cyclophorus appendiculatus* (Pfeiffer, 1854) **C** lectotype NHMUK 20130079/1, and **D** paralectotype NHMUK 20130079/2-3 **E, F** *Cyclophorus aquilus* (Sowerby I, 1843) **E** lectotype NHMUK 20110225/1, and **F** paralectotype NHMUK 2011225/2-3.

**Figure 4.**
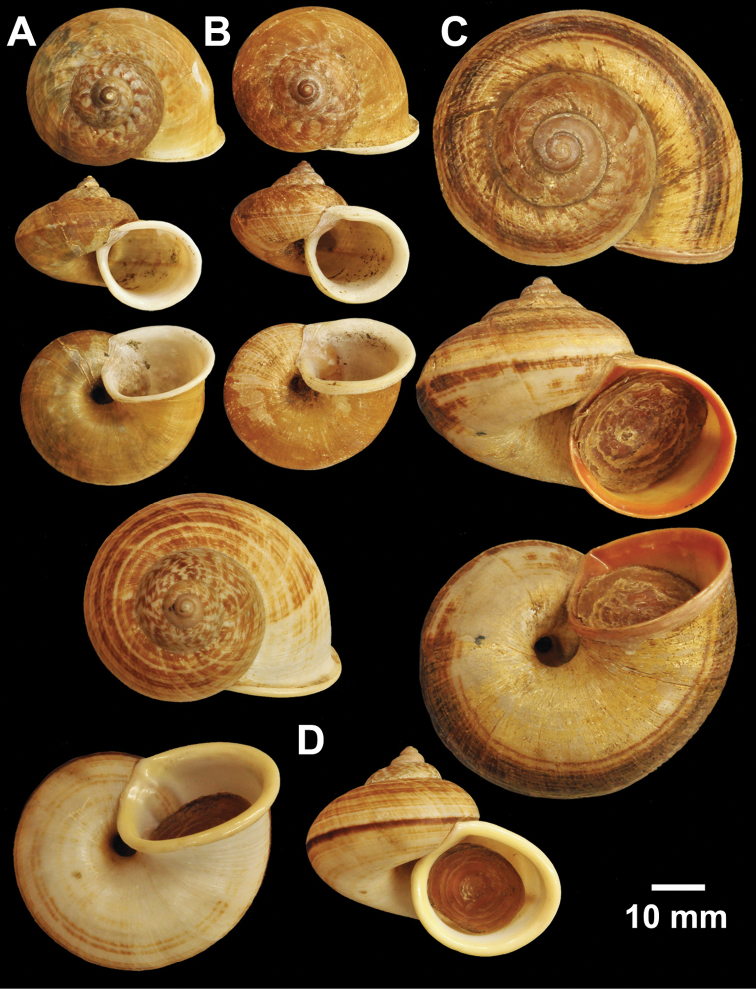
Types of *Cyclophorus* species. **A, B** *Cyclophorus bapuensis* Godwin-Austen, 1915 **A** lectotype NHMUK 1903.7.1.3108/1, and **B** paralectotype NHMUK 1903.7.1.3108/2-3 **C** *Cyclophorus beddomeanus* Preston, 1914 lectotype NHMUK 1936.4.15.22 **D** *Cyclophorus bensoni* (Pfeiffer, 1854) lectotype NHMUK 20130115.

**Figure 5.**
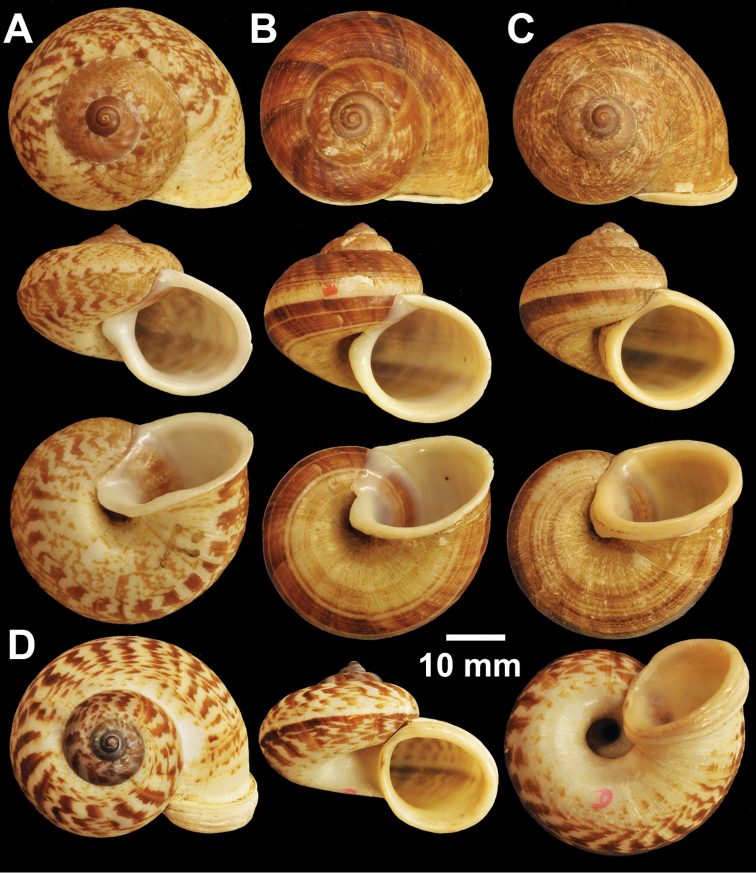
Types of *Cyclophorus* species. **A** *Cyclophorus cochranei* Godwin-Austen, 1889 lectotype NHMUK 1889.12.7.5 **B, C** *Cyclophorus consociatus* Smith, 1893 **B** lectotype NHMUK 1893.2.26.8, and **C** paralectotype NHMUK 1893.2.26.9-10 **D** *Cyclophorus crassalabella* Godwin-Austen, 1888 lectotype NHMUK 1911.6.10.8.

**Figure 6.**
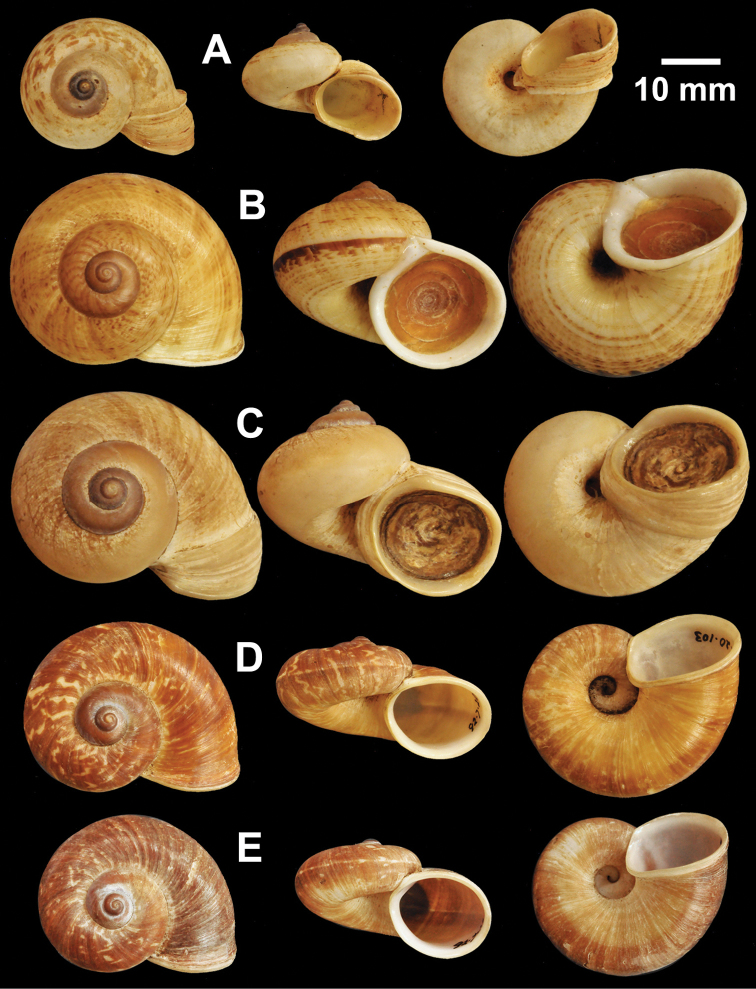
Types of *Cyclophorus* species. **A** *Cyclophorus cucullatus* (Gould, 1856) paralectotypes NHMUK 20130116 **B, C** *Cyclophorus eudeli* Smith, 1893 **B** lectotype NHMUK 1893.2.26.5, and **C** paralectotype NHMUK 1893.2.26.6-7 **D, E** *Cyclophorus everetti* Smith, 1892 **D** lectotype NHMUK 1892.7.20.103, and **E** paralectotype NHMUK 1892.7.23.1-2.

**Figure 7.**
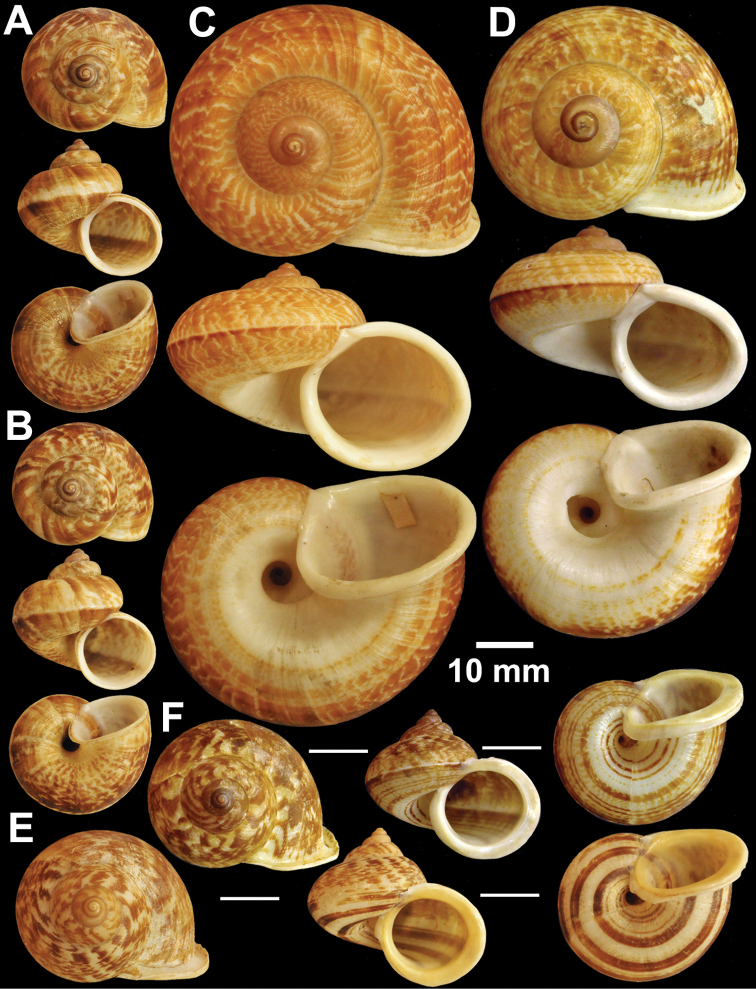
Types of *Cyclophorus* species. **A, B** *Cyclophorus exaltatus* (Pfeiffer, 1855) **A** lectotype NHMUK 1980041/1, and **B** paralectotype NHMUK 1980041/2-3 **C, D** *Cyclophorus excellens* (Pfeiffer, 1855) **C** lectotype NHMUK 20130084/1, and **D** paralectotype NHMUK 20130084/2 **E, F** *Cyclophorus expansus* (Pfeiffer, 1853) **E** lectotype NHMUK 20130086/1, and **F** paralectotype NHMUK 20130086/2-3.

**Figure 8.**
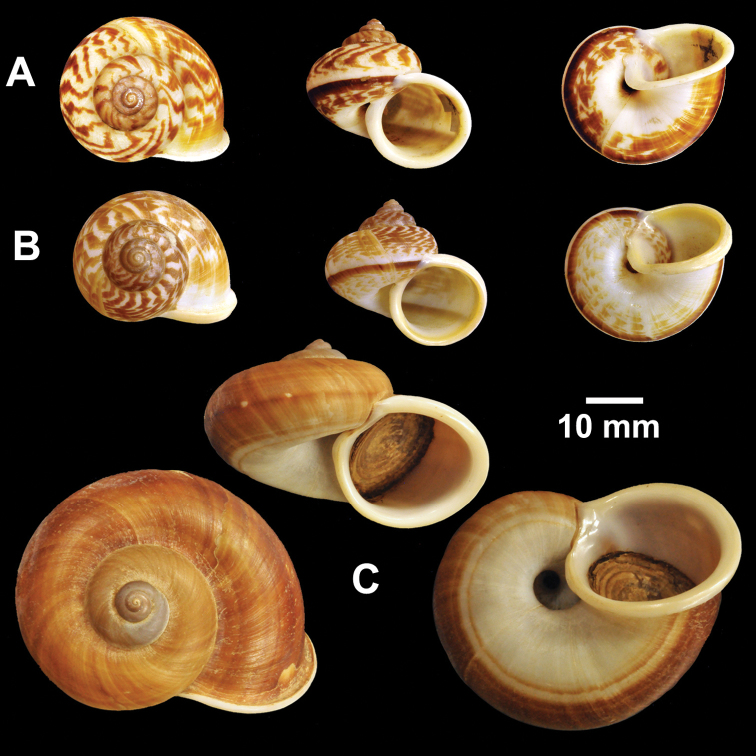
Types of *Cyclophorus* species. **A, B** *Cyclophorus fulguratus* (Pfeiffer, 1854) **A** lectotype NHMUK 20130117/1, and **B** paralectotype NHMUK 20130117/2-3 **C** *Cyclophorus fultoni* Godwin-Austen & Beddome, 1894, lectotype NHMUK 1894.6.20.1.

**Figure 9.**
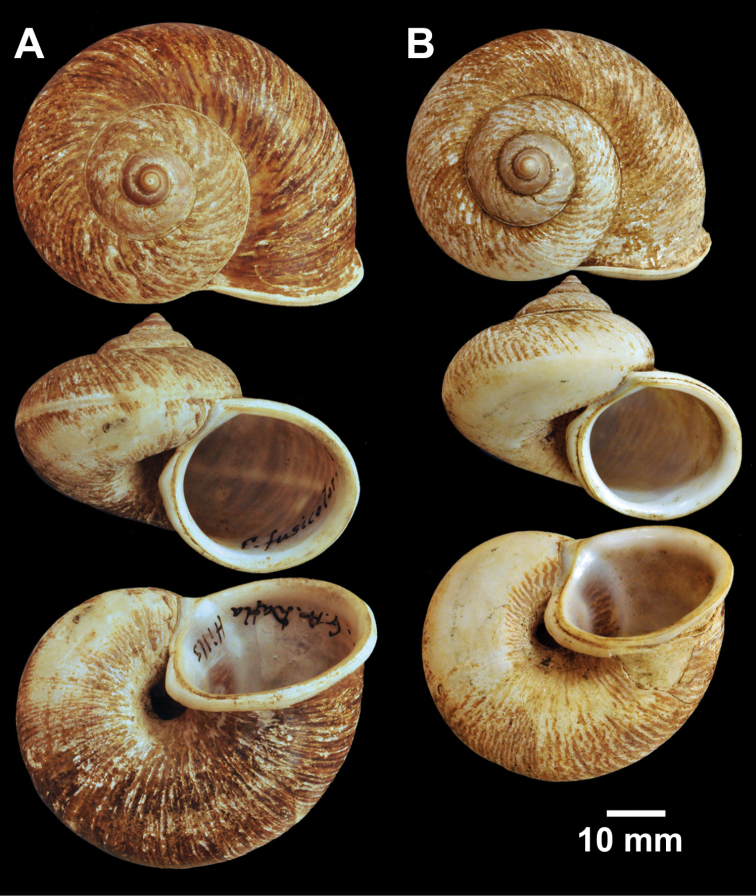
Types of *Cyclophorus* species. **A, B** *Cyclophorus fuscicolor* Godwin-Austen, 1876 **A** lectotype NHMUK 1903.7.1.1452/1, and **B** paralectotype NHMUK 1903.7.1.1452/2.

**Figure 10.**
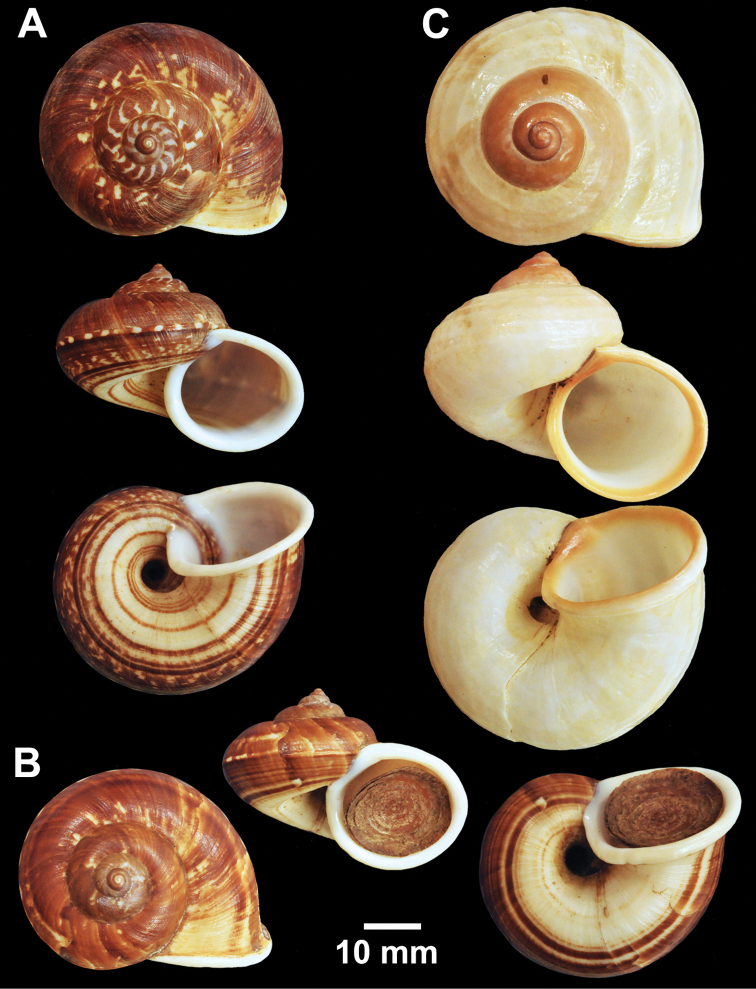
Types of *Cyclophorus* species. **A, B** *Cyclophorus haughtoni* Theobald, 1858 **A** lectotype NHMUK 1888.12.4.1953, and **B** paralectotypes NHMUK 1888.12.4.1954 **C** *Cyclophorus himalayanus* (Pfeiffer, 1853), lectotype NHMUK 20130118.

**Figure 11.**
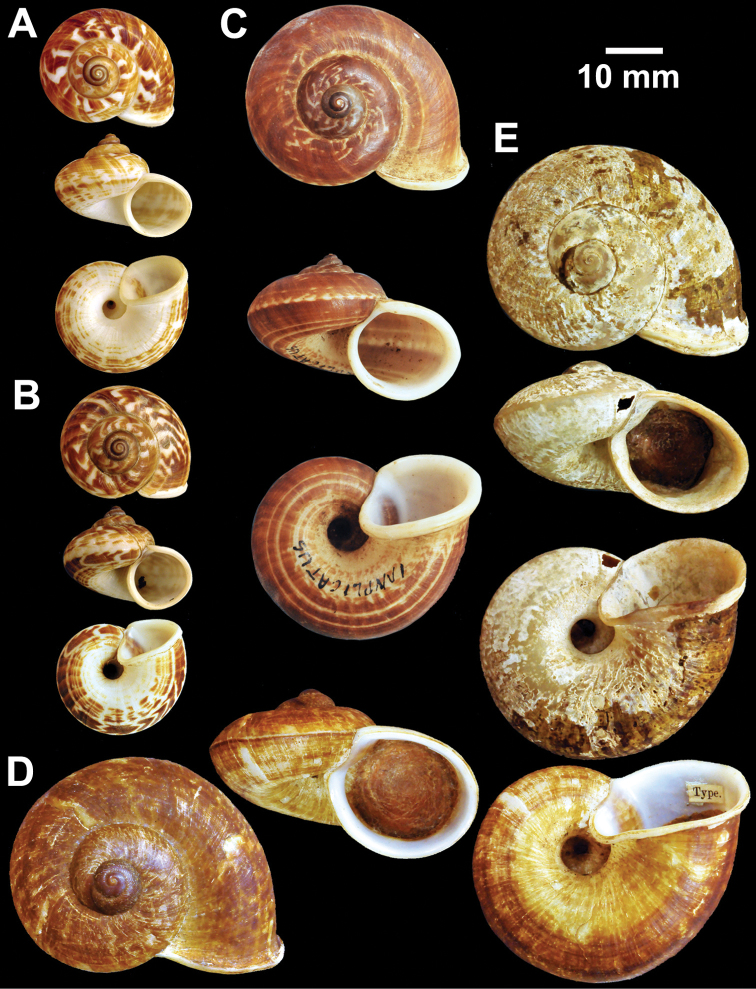
Types of *Cyclophorus* species. **A, B** *Cyclophorus ibyatensis* (Pfeiffer, 1854) **A** lectotype NHMUK 20130081/1, and **B** paralectotype NHMUK 20130081/2 **C** *Cyclophorus implicatus* Bavay and Dautzenberg, 1908, paralectotype NHMUK 20130087 **D, E** *Cyclophorus kinabaluensis* Smith, 1895 **D** lectotype NHMUK 1894.7.20.38, and **E** paralectotype NHMUK 1893.6.8.31.

**Figure 12.**
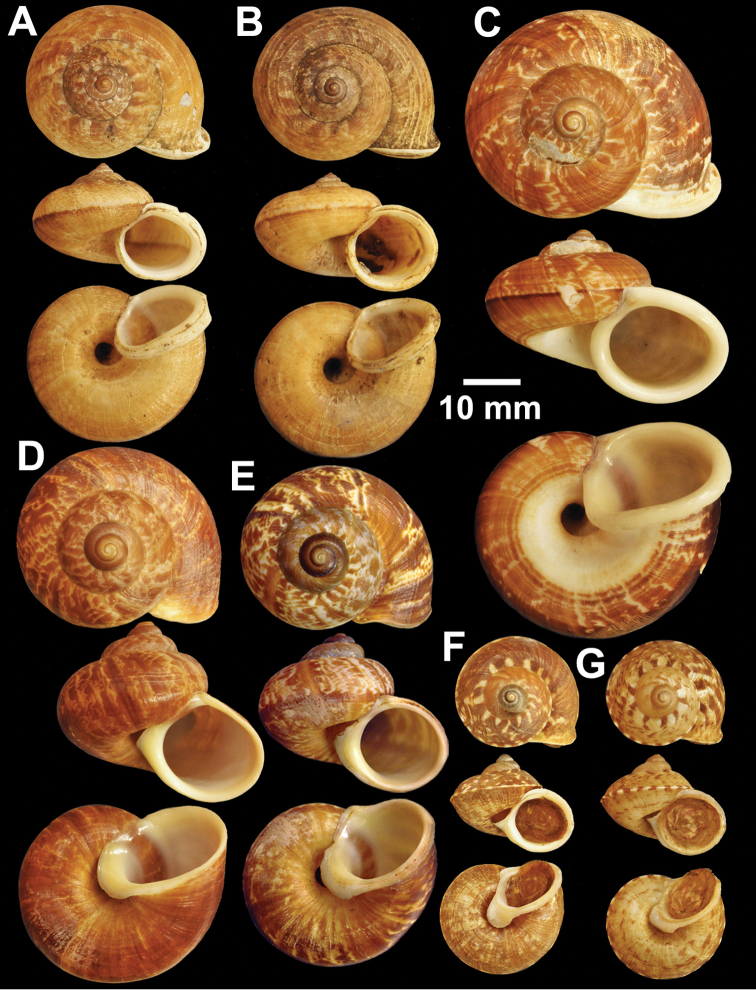
Types of *Cyclophorus* species. **A, B** *Cyclophorus koboensis* Godwin-Austen, 1915 **A** lectotype NHMUK 1903.7.1.3579/1, and **B** paralectotype NHMUK 1903.7.1.3579/2-4 **C** *Cyclophorus labiosus* (Pfeiffer, 1854), lectotype NHMUK 20130080 **D, E** *Cyclophorus linguiferus* (Sowerby I, 1843) **D** lectotype NHMUK 20110269/1, and **E** paralectotype NHMUK 20110269/2-3 **F, G** *Cyclophorus lingulatus* (Sowerby I, 1843) **F** lectotype NHMUK 20110272/1, and **G** paralectotype NHMUK 20110272/2-3.

**Figure 13.**
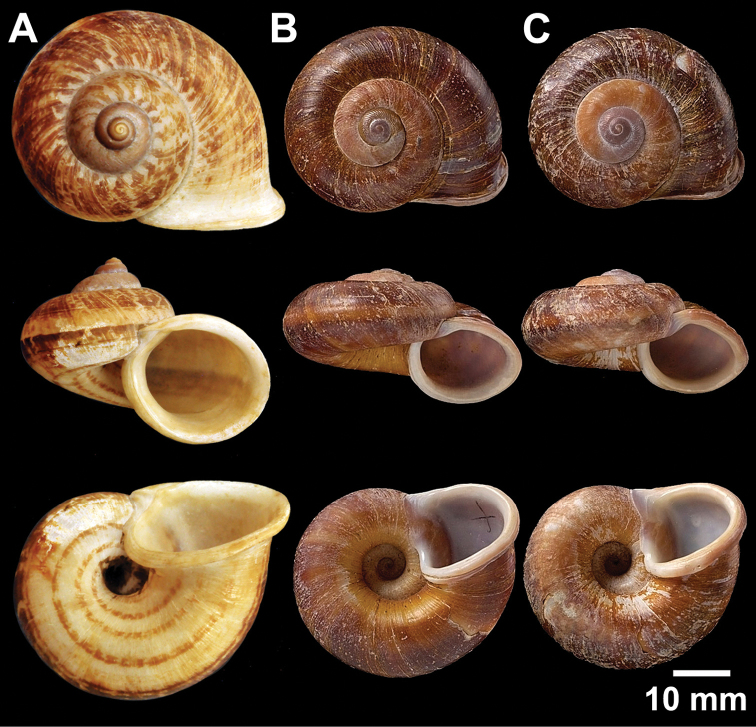
Types of *Cyclophorus* species. **A** *Cyclophorus malayanus* (Benson, 1852), syntype NHMUK 20130089 **B, C** *Cyclophorus monachus* (Morelet, 1866) **B** lectotype NHMUK 1893.2.4.499, and **C** paralectotype NHMUK 1893.2.4.500.

**Figure 14.**
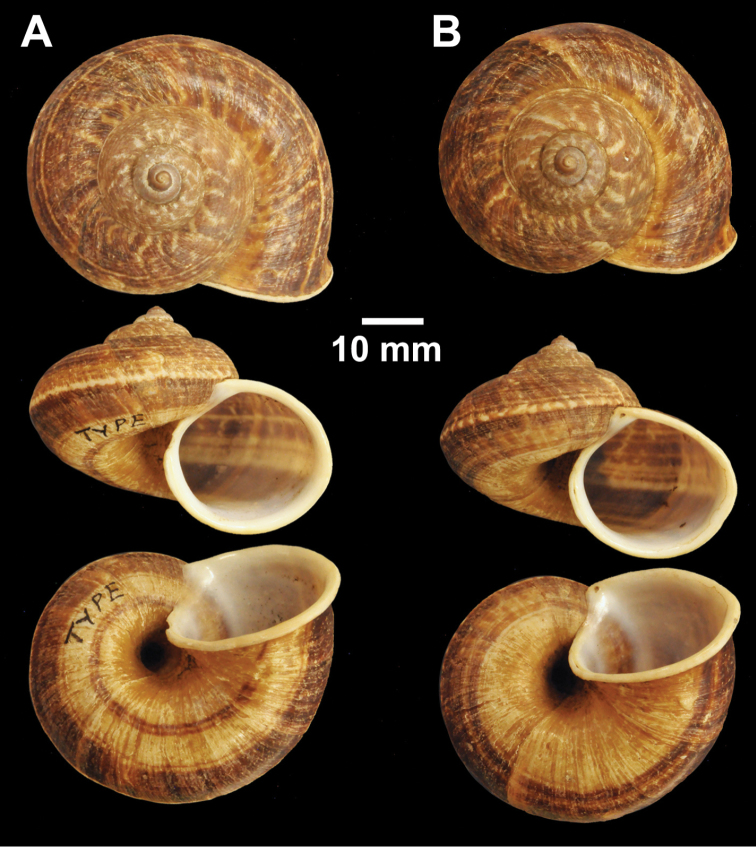
Types of *Cyclophorus* species. **A, B** *Cyclophorus muspratti* Godwin-Austen & Beddome, 1894 **A** holotype NHMUK 1903.7.1.1427/1, and **B** paratype NHMUK 1903.7.1.1427/2-4.

**Figure 15.**
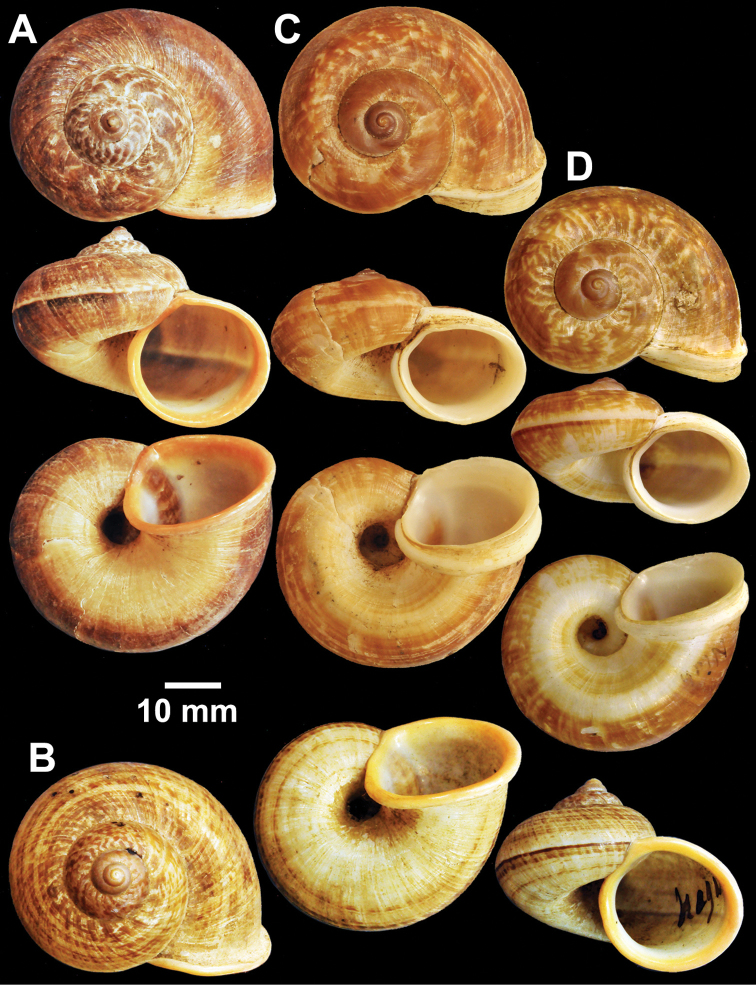
Types of *Cyclophorus* species. **A, B** *Cyclophorus nagaensis* Godwin-Austen & Beddome, 1894 **A** lectotype NHMUK 1903.7.1.1456/1, and **B** paralectotype NHMUK 1903.7.1.1456/2-4 **C, D** *Cyclophorus niahensis* Godwin-Austen, 1889 **C** lectotype NHMUK 1889.12.7.3, and **D** paralectotype NHMUK 1889.12.7.4.

**Figure 16.**
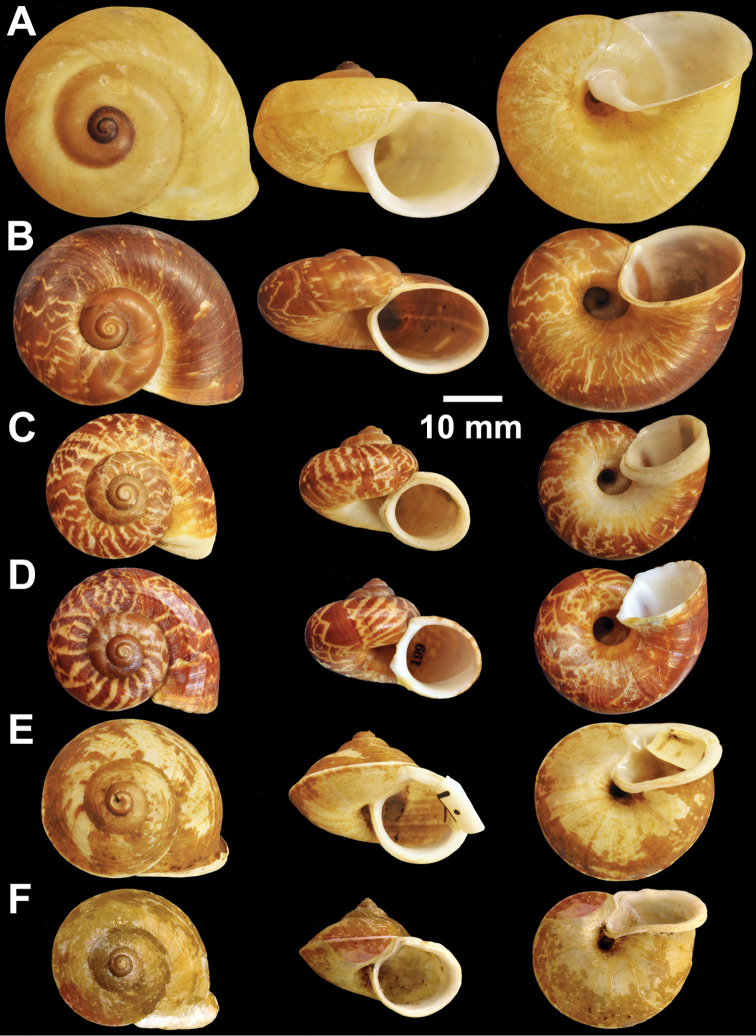
Types of *Cyclophorus* subspecies. **A** *Cyclophorus cochranei ochraceus* Godwin-Austen, 1889, lectotype NHMUK 1889.12.7.6. Types of *Cyclophorus* species **B** *Cyclophorus phlegethon* Godwin-Austen, 1889, holotype NHMUK 1998011 **C, D** *Cyclophorus picturatus* (Pfeiffer, 1854) **C** lectotype NHMUK 20130082/1, and **D** paralectotype NHMUK 20130082/2-3 **E, F** *Cyclophorus poeciloneurus* Godwin-Austen & Beddome, 1894 **E** lectotype NHMUK 1903.7.1.1522/1, and **F** paralectotype NHMUK 1903.7.1.1522/2.

**Figure 17.**
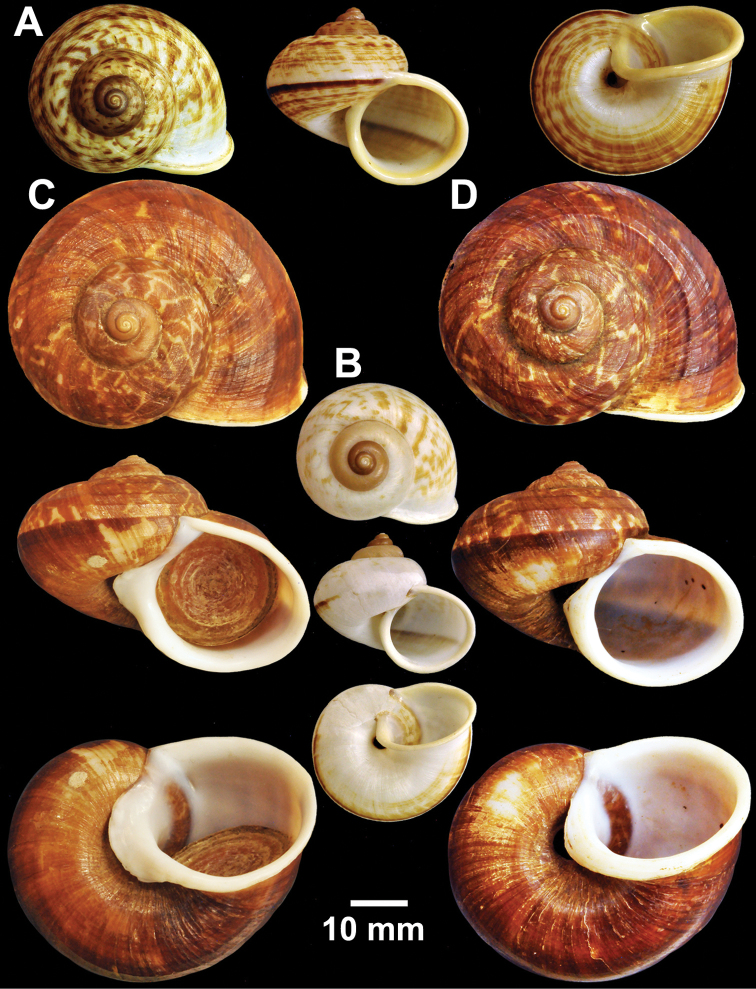
Types of *Cyclophorus* species. **A, B** *Cyclophorus fulguratus rangunensis* Kobelt, 1908 **A** lectotype NHMUK 20130091/1, and **B** paralectotype NHMUK 20130091/2-3 **C, D** *Cyclophorus eximus rouyeri* Bullen, 1906 **C** lectotype NHMUK 1906.1.16.51, and **D** paralectotype NHMUK 20130078.

**Figure 18.**
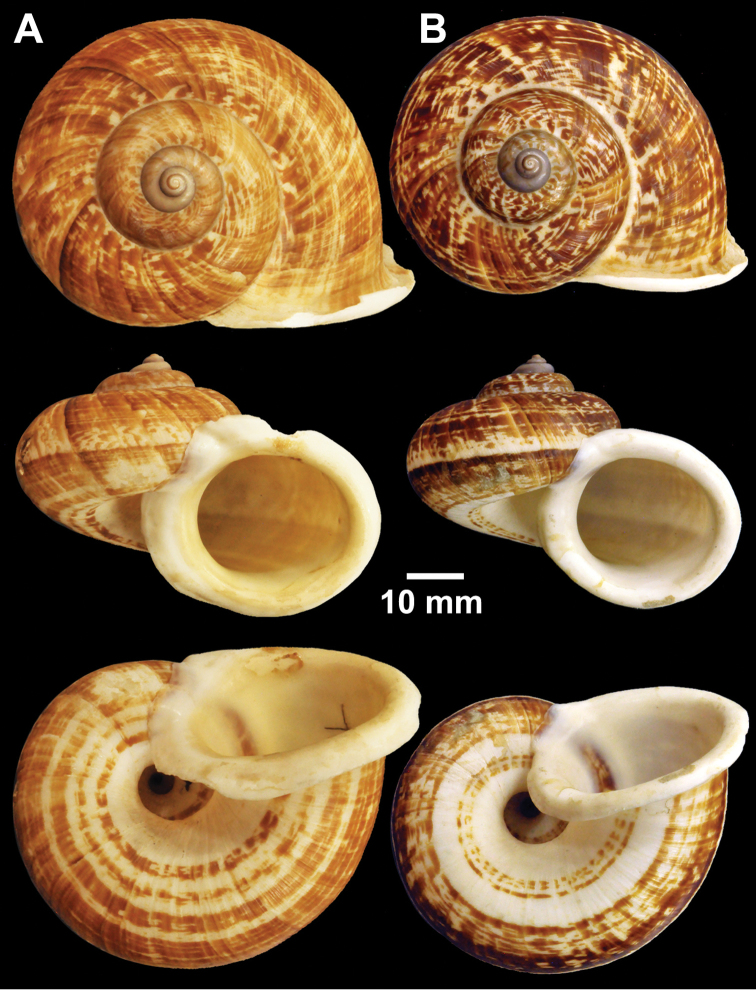
Types of *Cyclophorus* species. **A, B** *Cyclophorus saturnus* Pfeiffer, 1862 **A** lectotype NHMUK 20130119/1, and **B** paralectotype NHMUK 20130119/2-3.

**Figure 19.**
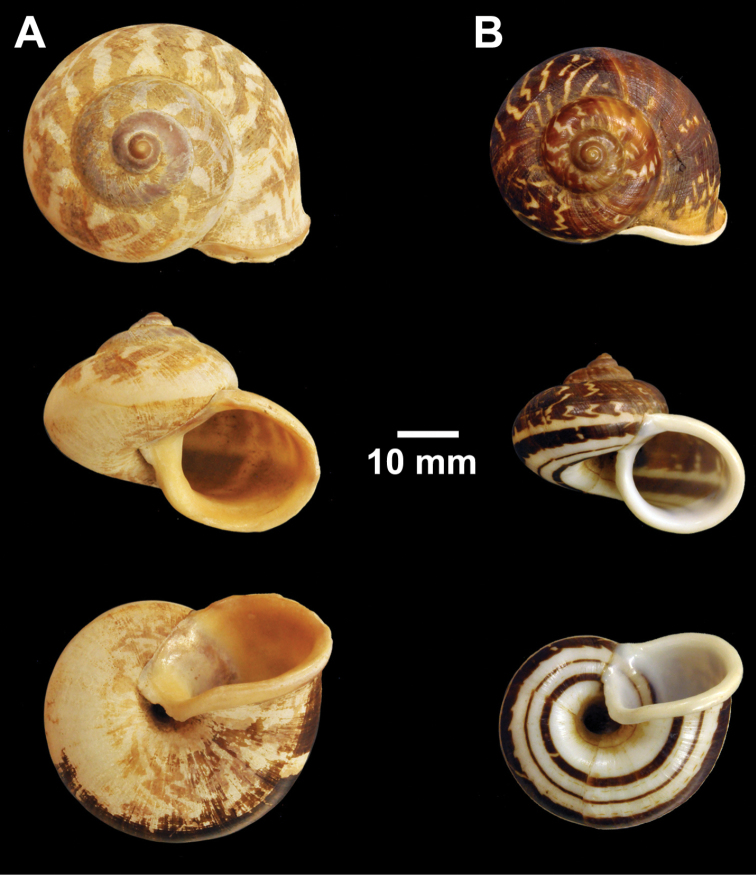
Types of *Cyclophorus* species. **A** *Cyclophorus schepmani* Laidlaw, 1957, paratype NHMUK 1957.11.18.7 **B** *Cyclophorus serratizona* Hanley and Theobald, 1876, possible syntypes NHMUK 88.12.4.1955.

**Figure 20.**
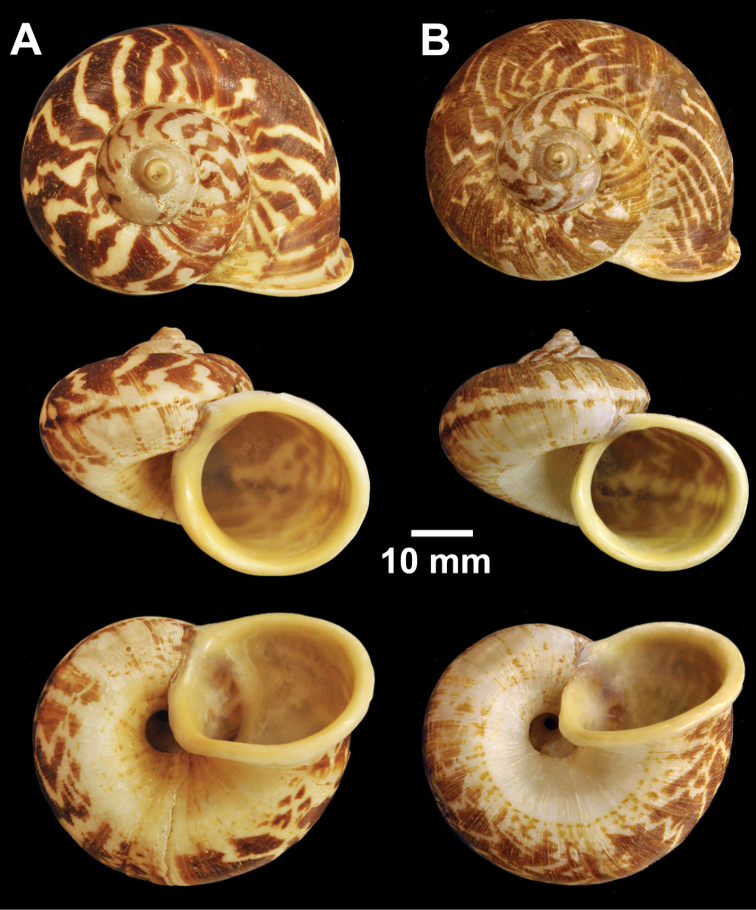
Types of *Cyclophorus* species. **A, B** *Cyclophorus siamensis* (Sowerby I, 1850) **A** lectotype NHMUK 20130088/1, and **B** paralectotype NHMUK 20130088/2.

**Figure 21.**
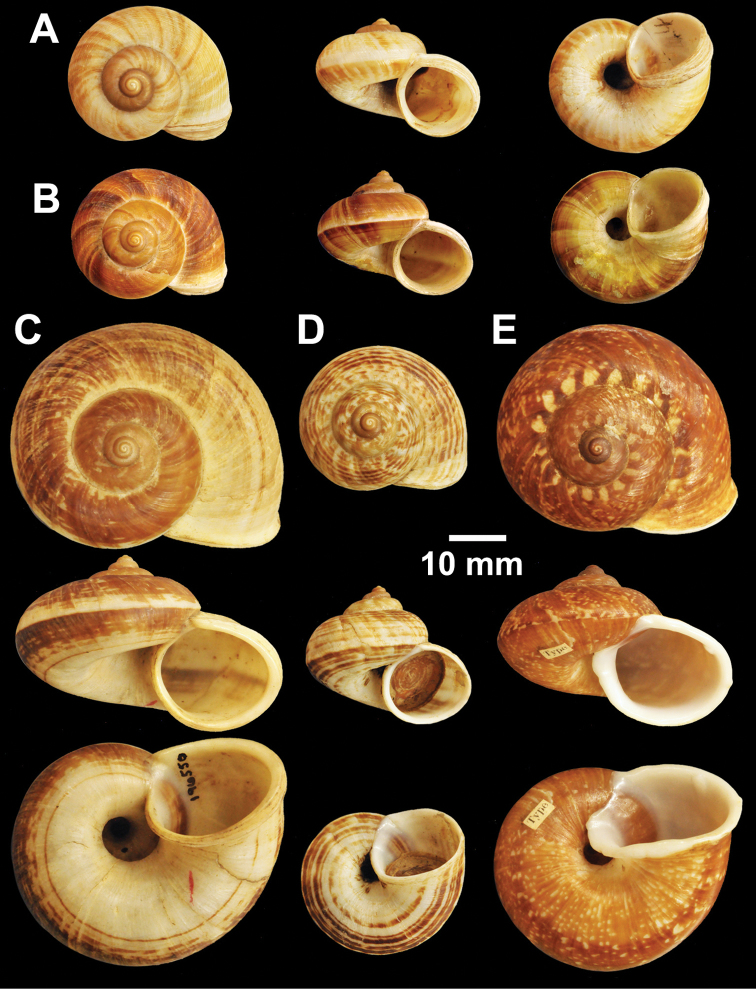
Types of *Cyclophorus* species. **A, B** *Cyclophorus spironema* (Pfeiffer, 1855) **A** lectotypes NHMUK 20130083/1, and **B** paralectotype NHMUK 20130083/2-3 **C** *Cyclophorus subblaevigatus* Blanford, 1869, lectotype NHMUK 196550 **D** *Cyclophorus taeniatus* (Pfeiffer, 1855), lectotype NHMUK 20130120 **E** *Cyclophorus talboti* Godwin-Austen, 1889, lectotype NHMUK 1889.12.7.7.

**Figure 22.**
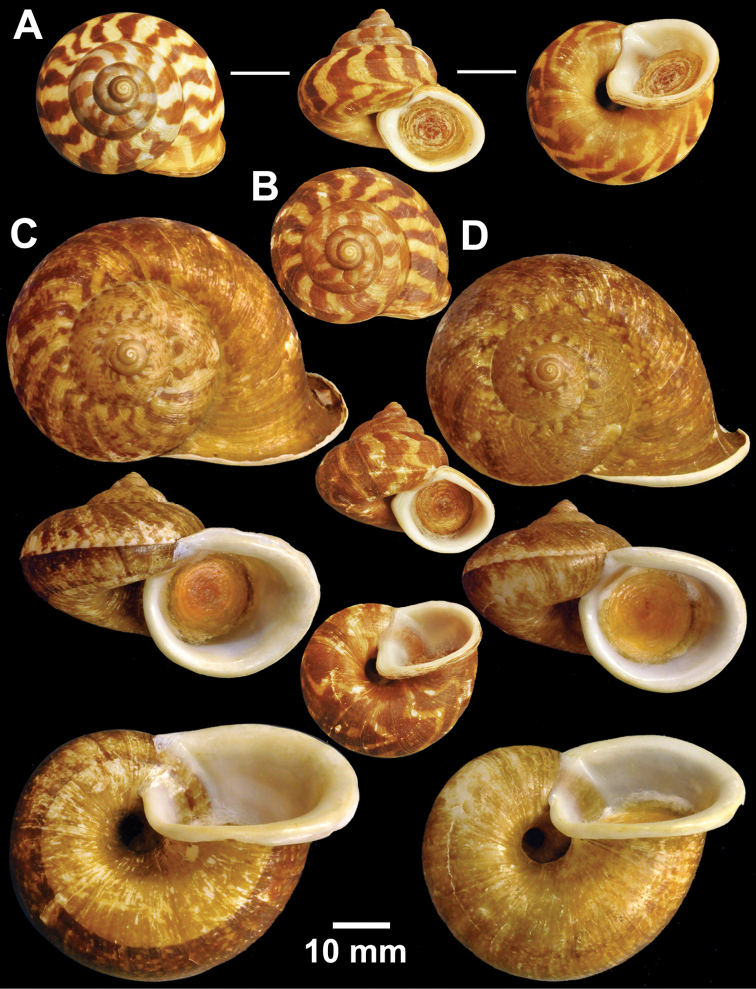
Types of *Cyclophorus* species. **A, B** *Cyclophorus tigrinus* (Sowerby I, 1843) **A** lectotype NHMUK 20110231/1, and **B** paralectotype NHMUK 20110231/2-3 **C, D** *Cyclophorus tuba* (Sowerby I, 1842) **C** lectotype NHMUK 20120064/1, and **D** paralectotype NHMUK 20120064/2.

**Figure 23.**
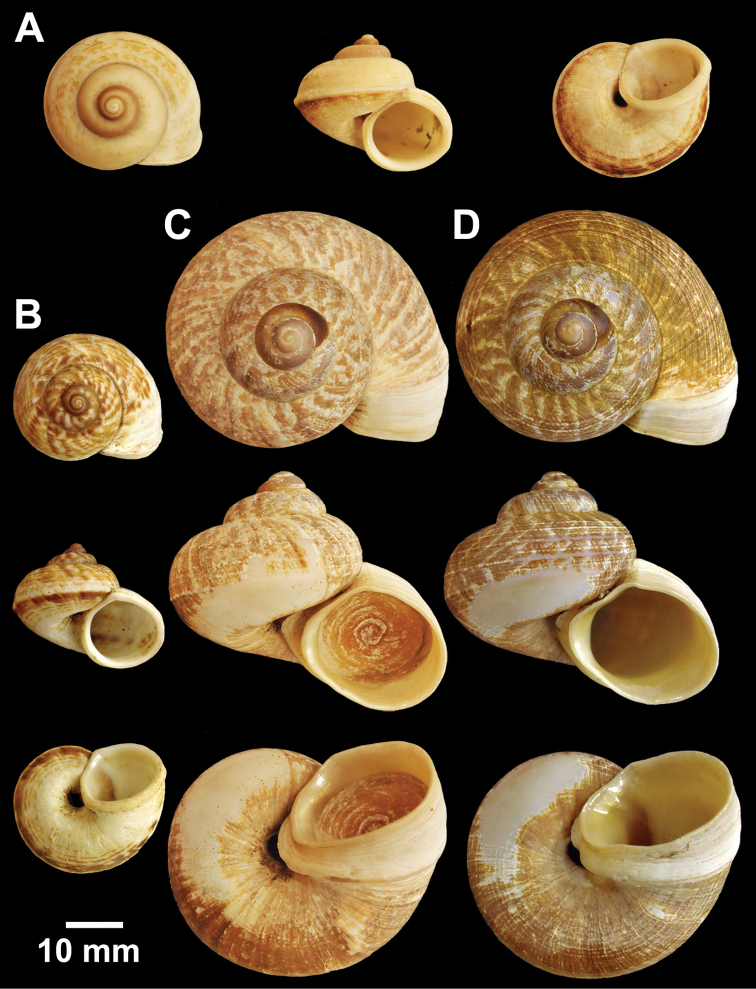
Types of *Cyclophorus* species. **A, B** *Cyclophorus turgidus* (Pfeiffer, 1851) **A** lectotype NHMUK 20040591/1, and **B** paralectotype NHMUK 20040591/2-3 **C, D** *Cyclophorus validus* (Sowerby I, 1842) **C** lectotype NHMUK 20110280/1, and **D** paralectotype NHMUK NHMUK 20110280/2-3.

## Supplementary Material

XML Treatment for Cyclophorus aborensis

XML Treatment for Cyclophorus affinis

XML Treatment for Cyclophorus amoenus

XML Treatment for Cyclophorus appendiculatus

XML Treatment for Cyclophorus aquilus

XML Treatment for Cyclophorus bapuensis

XML Treatment for Cyclophorus beddomeanus

XML Treatment for Cyclophorus bensoni

XML Treatment for Cyclophorus cochranei

XML Treatment for Cyclophorus consociatus

XML Treatment for Cyclophorus crassalabella

XML Treatment for Cyclophorus cucullata

XML Treatment for Cyclophorus eudeli

XML Treatment for Cyclophorus everetti

XML Treatment for Cyclophorus exaltatus

XML Treatment for Cyclophorus excellens

XML Treatment for Cyclophorus expansus

XML Treatment for Cyclophorus fulguratus

XML Treatment for Cyclophorus fultoni

XML Treatment for Cyclophorus fuscicolor

XML Treatment for Cyclophorus haughtoni

XML Treatment for Cyclophorus himalayanus

XML Treatment for Cyclophorus ibyatensis

XML Treatment for Cyclophorus implicatus

XML Treatment for Cyclophorus kinabaluensis

XML Treatment for Cyclophorus koboensis

XML Treatment for Cyclophorus labiosus

XML Treatment for Cyclophorus linguiferus

XML Treatment for Cyclophorus lingulatus

XML Treatment for Cyclophorus malayanus

XML Treatment for Cyclophorus monachus

XML Treatment for Cyclophorus muspratti

XML Treatment for Cyclophorus nagaensis

XML Treatment for Cyclophorus niahensis

XML Treatment for Cyclophorus cochranei ochraceus

XML Treatment for Cyclophorus phlegethon

XML Treatment for Cyclophorus picturatus

XML Treatment for Cyclophorus poeciloneurus

XML Treatment for Cyclophorus fulguratus rangunensis

XML Treatment for Cyclophorus eximus rouyeri

XML Treatment for Cyclophorus saturnus

XML Treatment for Cyclophorus schepmani

XML Treatment for Cyclophorus serratizona

XML Treatment for Cyclophorus siamensis

XML Treatment for Cyclophorus spironema

XML Treatment for Cyclophorus subblaevigatus

XML Treatment for Cyclophorus taeniatus

XML Treatment for Cyclophorus talboti

XML Treatment for Cyclophorus tigrinus

XML Treatment for Cyclophorus tuba

XML Treatment for Cyclophorus turgidus

XML Treatment for Cyclophorus validus
